# Amphiphilic Low-Molecular-Weight Gelators Bearing β-*S*-*N*-Acetylglucosamine Linked to a Tartaric Acid Scaffold: Synthesis, Self-Assembly and Wheat Germ Agglutinin Binding

**DOI:** 10.3390/gels10010005

**Published:** 2023-12-21

**Authors:** Vicente Leafar Peña García, Pablo Héctor Di Chenna, María Laura Uhrig

**Affiliations:** 1 Departamento de Química Orgánica, Facultad de Ciencias Exactas y Naturales, Universidad de Buenos Aires, Intendente Güiraldes 2160, 3er piso, Pabellón 2, Ciudad Universitaria, Buenos Aires C1428EGA, Argentina; vicentepena@qo.fcen.uba.ar; 2 Centro de Investigaciones en Hidratos de Carbono (CIHIDECAR), Consejo Nacional de Investigaciones Científicas y Técnicas (CONICET)–Universidad de Buenos Aires, Buenos Aires C1428EGA, Argentina; 3 Unidad de Microanálisis y Métodos Físicos en Química Orgánica (UMYMFOR), Consejo Nacional de Investigaciones Científicas y Técnicas (CONICET)–Universidad de Buenos Aires, Buenos Aires C1428EGA, Argentina

**Keywords:** supramolecular gel, amphiphile, carbohydrate, lectin, organogel, hydrogel

## Abstract

The self-assembly of carbohydrate-based amphiphiles can lead to colloidal soft materials such as supramolecular gels featuring highly desirable characteristics like biodegradability and biocompatibility. The report herein presents the synthesis, characterization and supramolecular self-assembly, physical gelation and wheat lectin binding of two structurally related amphiphilic compounds having β-*S*-*N*-acetylglucosamine residues linked to a 2,3-diacyl-*N*,*N*′-dipropargylated-l-tartaric diamide. A 1-thio-β-*N*-acetyl-d-glucosamine precursor attached to a conveniently functionalized linker with an azido group was synthesized by means of a one-pot procedure followed by deprotection. A click reaction successfully led to the two amphiphiles, which differed in length of the fatty acid attached to the tartaric acid scaffold. Although both compounds are poorly soluble in water and organic solvents, the difference in terms of hydrophilic moieties provided them with distinct supramolecular gelation properties. While the presence of an octadecyl chain produced a hydrogelator, the dodecadecyl homologue would only form weak gels in DMSO. SEM and rheology experiments confirmed the characteristic fibrillar morphology and viscoelastic properties, in agreement with the presence of physical gels. Both amphiphiles were able to interact reversibly with wheat germ agglutinin (WGA), a lectin that specifically recognizes GlcNAc residues, indicating a potential use in the food industry, as a gluten sensitivity manager, as well as in health-related industries, for example, for drug delivery systems.

## 1. Introduction

It is well known that the self-assembly of amphiphilic compounds is governed by a delicate balance between hydrophilic and hydrophobic interactions that are highly influenced by the polar nature of the environment provided by the solvent [1]. In water, for example, amphiphile hydrophobic segments tend to aggregate and minimize exposure to the aqueous environment, while the hydrophilic regions interact with water molecules to stabilize the overall assembly structure [2]. This interplay of forces drives the spontaneous formation of various self-assembled structures, such as micelles, inverse micelles and also supramolecular gels, which can be adjusted by modifying the molecular architecture and composition of the polar head, the non-polar tail and, eventually, a linker or scaffold which binds them together. Amphiphiles, where the polar head is based on carbohydrates, have called significant attention in the last decade due to their unique characteristics [2,3]: they have a highly polar head and are potentially biocompatible and biodegradable, while the carbohydrate itself comes from renewable sources. In addition, other properties, such as biological recognition, can provide additional relevant properties that may have highly interesting applications. For instance, carbohydrates can be specifically recognized by lectins, and such recognition events can trigger, in turn, biological events both in intra- or extra-cellular spaces. Pathogen binding and internalization, for example, are mediated by carbohydrate–protein interactions. Concerning this, it could be mentioned that the interplay between carbohydrates and proteins in cell adhesion, inflammation processes, tumor progression and glycoprotein folding has been extensively documented [4,5]. Thus, the development of sugar-based hydrogels, which can interact with lectins, is an attractive field due to their potential application as directed drug delivery supramolecular systems to treat several illnesses. In this respect, some promising approaches have been reported on targeted drug delivery systems, with the encapsulation of doxorubicin in supramolecular systems decorated with mannosyl residues, which are effectively internalized by breast cancer cells, causing cell death [6,7].

Due to their specific and reversible interaction with certain sugars, legume lectins have been extensively used as glycobiological tools [8,9,10,11]. Interestingly, they have been used as suitable substitutes for animal lectins to study carbohydrate–protein interactions and for the development of test systems. Among plant-origin lectins, wheat germ agglutinin (WGA) has caught special attention due to its ability to recognize GlcNAc residues [12]. GlcNAc is a ubiquitous sugar present in cell glycoconjugates. It is present in the N- and O-linked glycans attached to proteins and is a fundamental sugar of glycosaminoglycan chains. WGA interaction with this sugar occurs through multivalent contacts, leading to the widely known glycoside cluster effect [13], characterized by a dramatic enhancement of protein affinity for a certain ligand when presented in a cluster rather than alone. WGA is a heterodimeric carbohydrate-binding protein [14] having a total of eight carbohydrate-recognition domains for N-acetylglucosamine [12,15]. Reports on its specific toxicity and biomedical potential make it attractive for the development of anticancer, antibacterial, and antiviral strategies [12,16]. Also, it is certain that WGA is an antinutritional factor involved in several autoimmune gastrointestinal disorders such the celiac disease [17,18]. Our group has recently described several amphiphilic compounds containing GlcNAc and AllNAc based on aromatic scaffolds, which have interacted multivalently with the WGA [19].

Now, regarding self-assembled structures and materials produced from sugar-based low-molecular-weight amphiphiles, it is worth mentioning that supramolecular gels (SG) are of great interest due to their properties as soft materials. Among their relevant properties, these materials are reversible, thermo-responsive, viscoelastic and, thus, have been studied for various high-tech applications [20]. As thermo-reversible materials, it is possible to transition from gel to solution and vice versa in a reversible way at a given transition temperature (T_gel_). However, despite the increasing number of SG discovered (by serendipity or by structure-based design) and the great progress in the study of their self-assembly, the design of new supramolecular gelators based on small molecules is still a challenge [21,22,23]. SGs based on carbohydrate-headed amphipathic compounds rely on the fact that hydrogen bonding governs their self-assembly, especially in non-polar solvents [24]. Moreover, the presence of NHAc motifs contributes to the self-assembly process with a strong supramolecular synthon such as the amide group, capable of giving and accepting strong hydrogen bonds [25,26].

So, with the purpose of designing the above-mentioned compounds, we have explored the use of tartaric acid as an internal scaffold for the synthesis of amphiphilic compounds, in the search of more complex structures than those reported, based on simple hydrophobic chains derived from fatty acids as hydrophobic tails [27]. Indeed, tartaric acid is an excellent chiral building block given that the presence of two hydroxyl groups and two carboxylic acid groups allows for orthogonal functionalization. Also, its inherent chirality is a positive feature for the formation of chiral supramolecular assemblies [28] and the amide groups provide additional hydrogen-bonding motifs. Our group has previously reported two disaccharide-based amphiphiles bearing a thiolactose polar head on a di-lauroyl-l-tartaric scaffold with differences in spacer length [29]. The shorter one was able to form chiral hydrogels that showed variation in the chiral assembly upon sol-gel transition. Furthermore, these amphiphiles were able to bind to peanut agglutinin (PNA), a specific lectin that binds to lactose. Moreover, there are reports on the synthesis and properties of tartaric acid-derived Gemini-like and Bola-like amphiphiles, showing interesting properties as bactericides and antimicrobians [28,30]. Also, thermo-responsible chiral hydrogels were developed from catanionic surfactant mixtures made of diacyl-tartaric acids [31], as well as amphiphilic tartrate derivatives with transdermal permeation-enhancing activity of theophylline [32].

**Figure 1:**
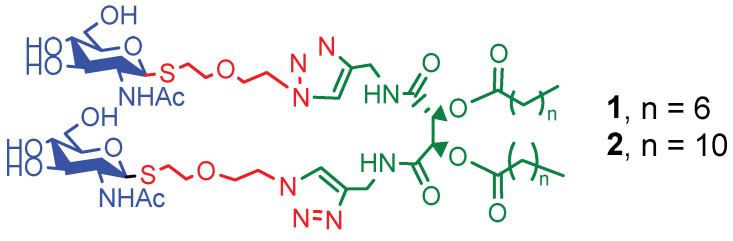
Amphiphilic compounds **1** and **2** synthesized and studied in this work. Polar head (blue), linker (red) and tartaric acid scaffold and non-polar tail (green).

Considering this background and based on our previously developed lactosyl-containing low-molecular-weight gelators as a starting point [29], we have designed amphiphiles **1** and **2** (Figure 1), exposing GlcNAc residues as potential hydrogelators that would be capable of binding to WGA selectively.

## 2. Results and Discussion

Considering the excellent results previously obtained with the tartaric acid scaffold to promote chiral self-assembly [29,33], we synthesized β-*S*-(*N*-acetyl)glucosamine derivatives to obtain amphiphiles that would be recognized by WGA and could also potentially self-assemble to produce supramolecular gels. In order to modulate the solubility (and self-assembly) in water and other organic solvents, the two amphiphiles differed in the length of the alkyl chain of the fatty acids, i.e., octanoic acid (caprylic acid, amphiphile **1**) and dodecanoic acid (lauric acid, amphiphile **2**).

### 2.1. Synthesis of Amphiphiles

**Scheme 1:**
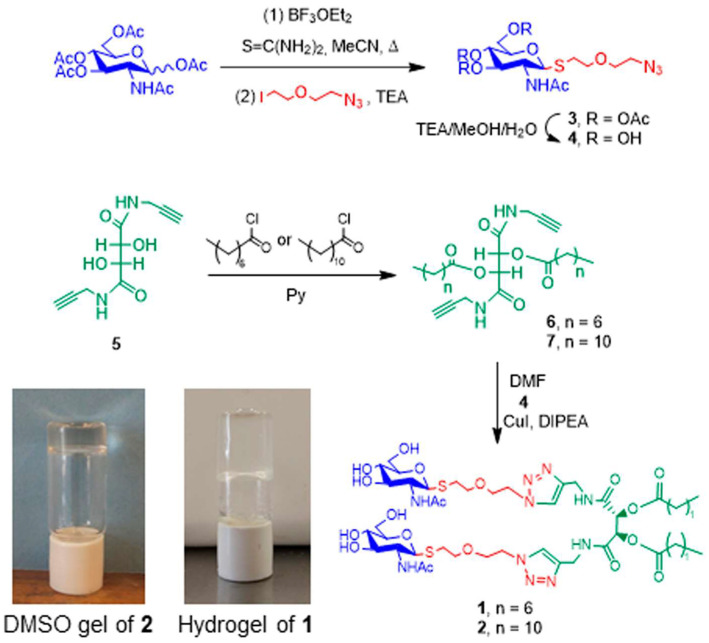
Synthesis of amphiphilic compounds **1** and **2**. The supramolecular gels of compound **1** in water (0.3 wt%) and compound **2** in DMSO (0.8 wt%) are shown. The hydrophobic portion was depicted in green, the sugar moiety in blue and the spacer in red.

The synthesis of supramolecular gelators **1** and **2** was performed as shown in Scheme 1. A 1-thio-β-*N*-acetyl-d-glucosamine precursor (**4**) attached to a linker conveniently functionalized with an azido group was synthesized using a one-pot procedure followed by deprotection (Scheme 1) as previously described [29,34]. Then, two diacyl *N*,*N*-dipropargylated tartaric acid scaffolds derived from caprylic acid and lauric acid, respectively, (compounds **6** and **7**) were prepared with the standard acylation of **5**. The polar head exposing the glucosamine residue was then connected to the l-tartaric acid scaffolds using click chemistry. The reaction between compound **7** and precursor **4** led to a viscous mixture which was difficult to manipulate. TLC analysis was not conclusive at first to determine whether the reaction was completed or not, thus, a fraction was acetylated under standard conditions. A single spot of R*f* = 0.71 (EtOAc/MeOH 7:3), corresponding to the acetylated derivative of compound **2** was observed. MS-ESI confirmed the presence of the product as a peak at *m*/*z* = 1563.7144, consistent with the formula C_70_H_112_N_10_NaO_24_S_2_. This result was encouraging, as it indicated that the click reaction had successfully occurred. The reaction was repeated, and then the purification of compounds **1** and **2** was attempted to overcome the challenge posed by the low solubility of compounds **1** and **2** in several solvent mixtures, given their amphiphilic nature. Finally, they were successfully purified using silica gel 60-column chromatography using a gradient of EtOAc:MeOH (80:20) → (50:50), containing 1–10% Pyridine. Compound **1**: R*_f_* = 0.4 (EtOAc:MeOH:Py 7:3:1%); compound **2**: R*f* = 0.2 (EtOAc:MeOH:Py 7:3:1%). The [M+H]^+^ values in the MS-ESI spectra of compounds **1** and **2** observed at *m*/*z* = 1177.548 and 1289.673 agreed with the pursued structures. Thus, this protocol provided, once again, interesting amphiphilic molecules with well-defined hydrophilic and hydrophobic parts connected using click chemistry and with the potential of also establishing strong hydrogen bonds through two amide groups.

Despite the complexity of the spectra of compounds **1** and **2**, all of the signals observed in both the ^1^H and ^13^C NMR spectra in DMSO-*d*_6_ could be unambiguously assigned with the assistance of bidimensional experiments. The spectra also confirmed the high purity of the products obtained after silica gel chromatography. In the ^1^H NMR spectra, the signals corresponding to the sugar moieties, the hydrophobic tails and the spacer were observed. The major difference noticed between the two compounds was the relative integration of the aliphatic protons, characteristics from the fatty acid residues. Among the diagnostic signals, two deshielded signals at ≈8.60 ppm (as a triplet) and 7.70 ppm (as a doublet) accounted for the amide protons of the tartaramide scaffolds and the GlcNAc residues, respectively, while a singlet at ≈7.80 corresponded to the triazole proton. The detection of a singlet at 5.50 ppm was diagnostic of the presence of the tartrate scaffold. It should be mentioned that both compounds **1** and **2** are symmetric, so the detection of a unique singlet in this region was indicative of product purity, as impurities due to structurally related compounds cause the appearance of extra signals. Also, the anomeric protons of the β-configured GlcNAc residues appeared at 4.34 ppm (as a doublet with *J* = 10.3 Hz) and were more protected than those observed for common O-glycosides, because of the effect of the sulfur atom. The diastereotopic hydrogens of the diethylene glycol spacer showed complex patterns mostly overlapped at ≈3.50–4.00 ppm, except for those linked to the sulfur carbon, appearing as nice multiplets in the range of 2.50–2.80 ppm.

### 2.2. Gelation Scope and Characterization of Supramolecular Gels

The supramolecular gelation scope and thermic stability of the gels obtained were studied using the standard inverted tube method [35] on 10 common solvents spanning from water to non-polar solvents, and mixtures of water and alcohols (see Table 1). At room temperature, the amphiphiles were insoluble in all of the solvents and mixtures tested.

The inverted tube test showed that, while amphiphile **1**, with the short alkyl chain, formed a stable gel in water with a minimal concentration for gelation (MCG) of 0.3 wt% (T_gel_ 34 °C), amphiphile **2** was insoluble in water, and could only gelate dimethyl sulfoxide (DMSO) with an MCG of 0.8 wt% (T_gel_ 52 °C; see Figures S2 and S3). The plot of T_gel_ vs. concentration showed the typical behavior; as the concentration increased, the T_gel_ increased for both gelators until a maximum T_gel_ of 63 °C was reached for both gels at a concentration of around 2.5 wt%. These results indicate that compound **1** is a much more efficient gelator since less mass is needed to prepare a stable gel in water (fewer molecules of gelator are needed to immobilize the same amount of solvent).

**Figure 2:**
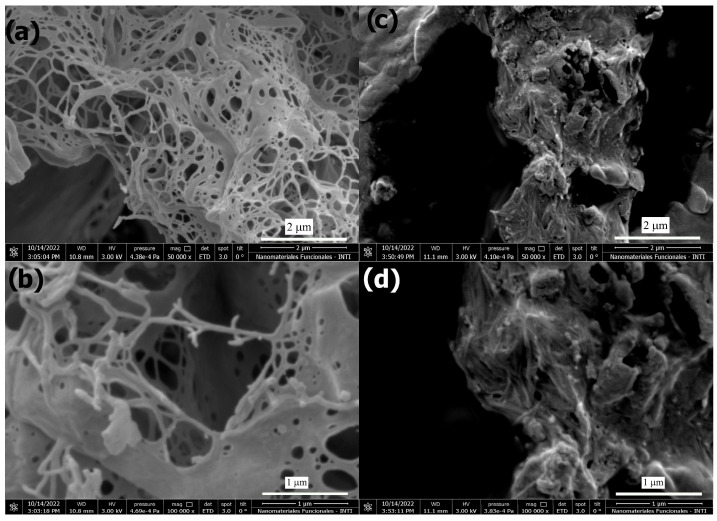
SEM images of the xerogels obtained from the hydrogel of amphiphile **1** (**a**,**b**) and from the DMSO gel of amphiphile **2** (**c**,**d**). Full-size images can be found at the Supplementary Material File (Figure S6).

**Figure 3:**
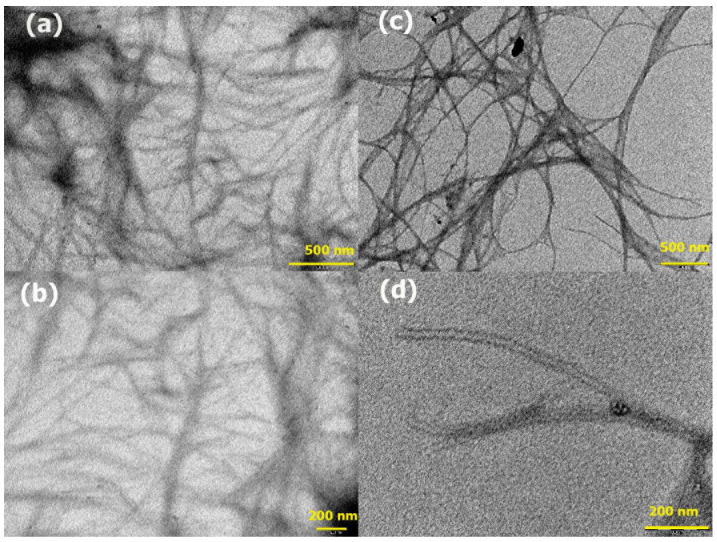
TEM images of the xerogels obtained from the hydrogel of amphiphile **1** (**a**,**b**) and from the DMSO gel of amphiphile **2** (**c**,**d**).

The morphology of the gels was studied by observing the scanning electron microscopy (SEM) and transmission electron microscopy (TEM) images taken on the xerogels (Figure 2 and Figure 3, respectively). Using lyophilization, the hydrogel of amphiphile **1** rendered a xerogel with a typical crossed-linked fibrillar network with fibers 33–45 nm wide and tens of micrometers long (Figure 2a,b). The DMSO gel of amphiphile **2** needed, due to the high boiling point of DMSO, a long time to dry off and, as shown in the SEM images, the structure of the fibrillar network collapsed completely, showing a compact, cross-linked network of fibers. Despite this, the fibers were visible in some areas and could be measured to be, on average, 35–46 nm wide and several microns long (Figure 2c,d).

TEM images also showed the typical cross-linked fibrillar networks (Figure 3), but in this case, the morphology of the fibers present in the xerogel of amphiphile **2** could be clearly observed (Figure 3c,d). In this case, it was possible to observe tubular fibers with an external diameter of 29–32 nm and an internal diameter of 11–12 nm (cf. Figure S7). This accounts for a shell width of around 10 nm. The length of a minimized single elongated molecule of amphiphile **2** (AM1) is around 2.6–2.8 nm, so these tubes may be multi-layered tubular fibers (worm-like micelles), a usual assembly formed by this kind of amphiphile [29]. For xerogel **1**, the cross-linked solid fibers had a diameter of 24–31 nm. In both cases, the fibers observed by TEM were thinner than the ones observed by SEM; the evaporation of the solvent from a very thin layer of gel on the grids seems to have affected the microstructure of the xerogel to a lesser extent. The width of the fibers observed using TEM also agree with the length of the molecules, the shorter gelator **1** showed thinner fibers than the larger gelator **2**. The fact that the SEM images showed a similar fiber width for gelator **1** and gelator **2** may be due to a collapsed fibrillar structure obtained with the lyophilization of the bulk gels.

To gain information about the crystallinity and the long-range order of the assemblies, powder X-ray diffraction experiments were performed on the xerogels of amphiphiles **1** and **2**. At small angles, no clear X-ray patterns were observed; only at wider angles (2θ 17–25°) a wide and flat peak related to the methylene-methylene distances (approx. 4.5 Å) of the aliphatic chains was visible (see Figure S5).

**Figure 4:**
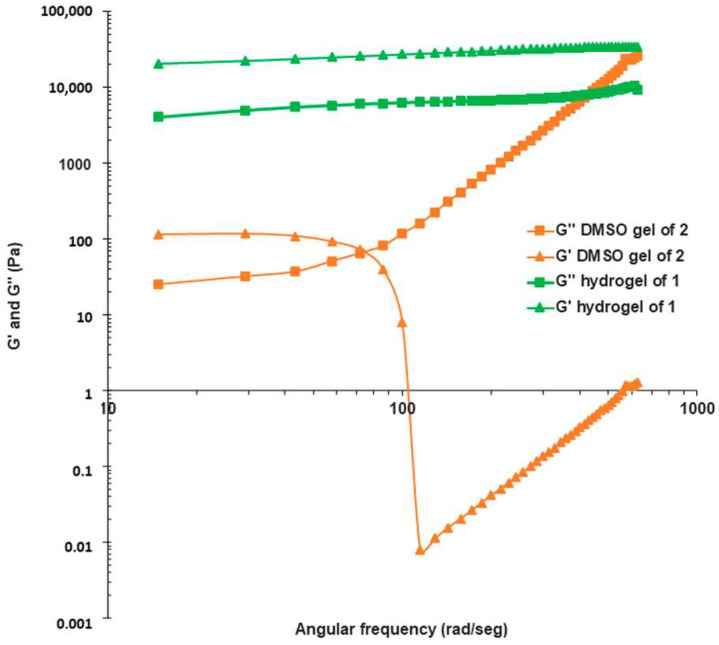
Dynamic frequency sweep experiment performed on a 0.8 wt% hydrogel of amphiphile **1** and a 0.8 wt% DMSO gel of amphiphile **2**.

To assess the viscoelastic nature and mechanical properties of the gels, rheological dynamic strain sweep and dynamic frequency sweep experiments were carried out in order to determine the linear viscoelastic region at room temperature (LVR, 1%; Figure S4 in Supplementary Materials). Thus, the hydrogel sample of amphiphile **1** behaved as a viscoelastic material and showed typical gel-like mechanical characteristics. The values of the sample dynamic storage modulus (G′) were higher by almost one order of magnitude than the corresponding dynamic loss modulus (G″), and both moduli showed a reduced dependence on frequency in the studied range (Figure 4) [36].

Additionally, for the DMSO gel of amphiphile **2**, both the G′ and G″ values showed a very short LVR, and the gel was destroyed at a very low frequency. In conclusion, while the hydrogel of amphiphile **1** behaved mechanically as a supramolecular gel over the whole LVR studied, the DMSO gel showed to be mechanically weak. This fact had already been observed while the gels were manipulated on the bench, being the DMSO gel formed by amphiphile 2 much more delicate and sensitive to shaking and mechanical perturbations, as it could be a cut by means of a spatula.

### 2.3. Circular Dichroism and Chirality of the Assemblies

**Figure 5:**
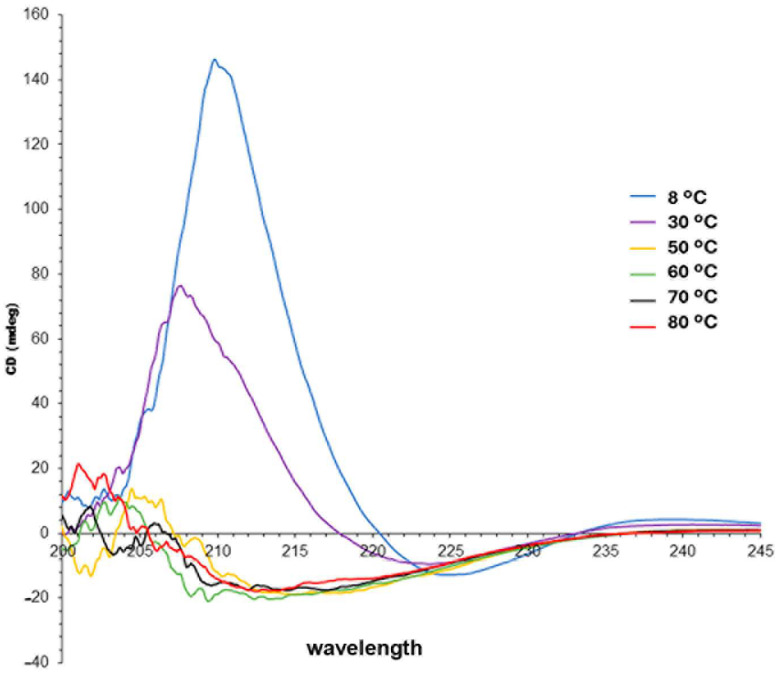
Temperature-dependent CD spectra of the hydrogel of **1** (0.3 wt%).

In order to study the chirality of the self-assembled aggregates of the amphiphiles and the sol-gel transition, Circular Dichroism (CD) spectra were performed at different temperatures (Figure 5). It is worth noticing that the amphiphiles only differed in the length of the alkyl chain (non-polar tail); they have the same chromophores, i.e., amide and triazole groups (with *n*–π* and π–π* transitions that absorb at about 210 nm) and the same chiral backbones, i.e., l-tartaric acid and glucose, so their CD spectra could be compared in a straightforward way.

Water has a UV cutoff wavelength of 190 nm but, unfortunately, DMSO has a UV cutoff wavelength of 268 nm, so the CD spectra for the DMSO gel of amphiphile **2** could not be measured. The hydrogel of amphiphile **1** showed a strong positive CD sign at 210 nm (+145 mDeg) and, as the temperature was raised, the CD sign decreased until becoming slightly negative above 50 °C. This may be accounting for the loss of the chiral assembly or a change to a different chiral assembly. In conclusion, amphiphile **1** forms an ordered chiral self-assembly compatible with a strong CD sign that changes from a high positive ellipticity to a low negative ellipticity after the T_gel_ is reached. Below 205 nm, a noisy and weak CD signal is observed due to electronic noise near the cutoff wavelength of water.

### 2.4. Wheat Germ Lectin-Binding Assay

**Figure 6:**
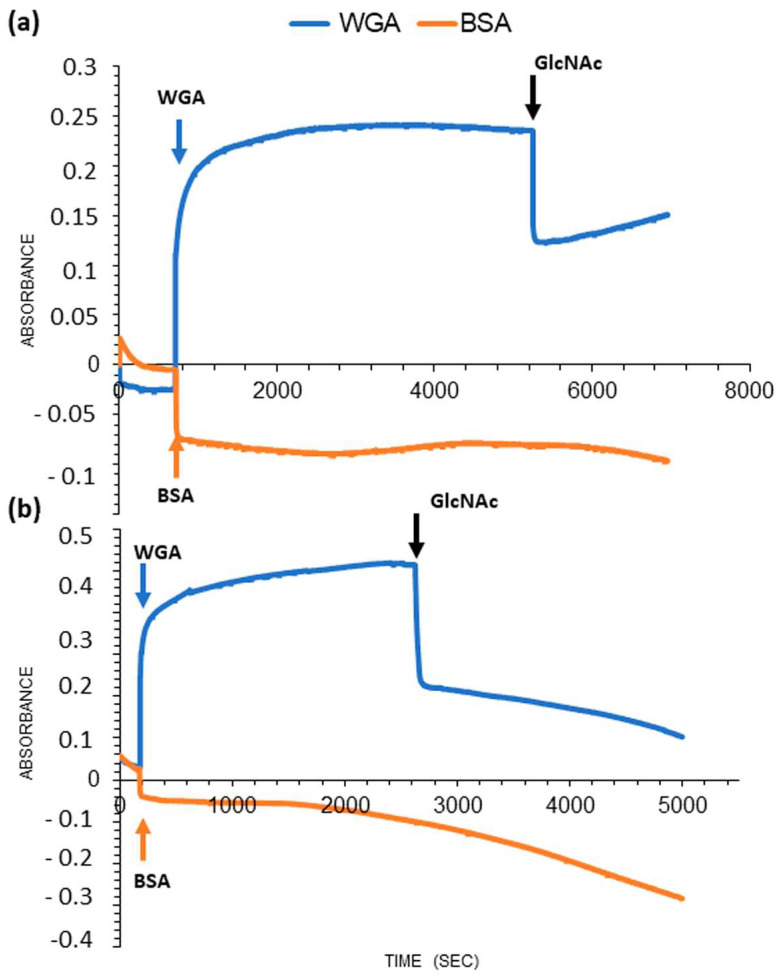
Turbidity assay of **1** (**a**) (7.07 × 10^−4^ M) or **2** (**b**) (8.67 × 10^−4^ M) at 677 nm in PBS buffer (pH = 7.4, NaH_2_PO_4_ 2.3 mM, Na_2_HPO_4_·2H_2_O 10 mM, NaCl 150.2 mM). At *t* = 4 min (**a**) and 2 min (**b**), a solution of WGA (7.6 × 10^−5^ M, blue line) or BSA (6.0 x 10^−5^ M, orange line) in the same buffer was added. At *t* = 35 min, a solution of *N*-acetyl-D-glucosamine (GlcNAc, 9.0 × 10^−2^ M, 30 µL) was added.

Lectins can reversibly recognize mono- and oligosaccharides with high specificity. They typically contain one carbohydrate recognition domain per monomer, and, via interaction with multivalent carbohydrate systems, they usually form cross-linked networks [19]. This multivalent assembly process is associated with a high affinity increase, the so-called cluster effect [14,37]. These typical multivalent cross-linking phenomena of lectins can be inhibited by adding an excess of the sugar ligand, for which the lectins are specific. In order to verify if amphiphiles **1** and **2** were able to form cross-linked networks via interaction with the *N*-acetylglucosamine-recognizing WGA lectin, a standard spectrophotometric turbidity assay was performed (Figure 6) [38,39].

As it can be seen in Figure 6a,b, upon adding WGA to a solution of compounds **1** or **2** in a PBS buffer, as described in the experimental section, an absorbance increase at 677 nm was observed, because of the turbidity associated with the crosslinking process upon the interaction between the self-assembled amphiphiles (**1** and **2**) and the heterodimeric WGA lectin. Upon further addition of a large excess of *N*-acetyl-d-glucosamine, a turbidity decrease was observed. This result shows that compounds **1** and **2** were displaced from the lectin recognition site by the free *N*-acetyl-d-glucosamine added. For both amphiphiles, a control experiment was performed with bovine serum albumin (BSA) and, as expected, it did not show any turbidity increase.

## 3. Conclusions

Multivalent carbohydrates, such as the amphiphiles **1** and **2** herein studied, are of significant interest for a wide range of biomedical applications such as immunotherapy, drug delivery systems, etc. A click reaction successfully led to the two amphiphiles, which differed in the length of the fatty acid attached to positions 2 and 3 of the tartaric acid scaffold. Although both compounds were found to be highly insoluble in many solvent systems, the difference in the hydrophilic moiety provided distinct gelation properties. Our results showed that the amphiphile with the short non-polar chain (**1**) has optimal solubility to self-assemble in water and form a stable hydrogel, with minimal concentration for gelation of 0.3 wt%. The hydrogel showed to self-assembly in a chiral arrangement as shown in the CD spectra with a strong positive CD sign that disappears upon heating. A longer chain rendered a highly hydrophobic amphiphile (**2**) poorly soluble in water, but that could form weak gels in DMSO at higher concentrations (MCG 0.8 wt%). This DMSO gel was less stable to thermal and mechanical stimuli. This could be related to a poorer cross-linked fibrillar network compared to amphiphile **1**. SEM microscopy of both xerogels confirmed the presence of cross-linked fibrillar networks as a consequence of their self-assembly. TEM images confirmed the presence of multilayered tubular micelles in the DMSO gel of amphiphile **2**. The fiber arrangement formed by compounds **1** and **2** does not hamper sugar epitope interaction with a *N*-acetyl-d-glucosamine-specific binding protein, the WGA lectin. Thus, a cross-linked system could be detected using turbidimetry and recognition of the sugar moiety by the protein occurs in a reversible way, given that the addition of an excess of *N*-acetyl-d-glucosamine disrupts the cluster. This study is a contribution to the knowledge of structural parameters impacting the self-assembly of sugar-based amphiphiles, a crucial step for the rational design and synthesis of nanostructures exposing specific sugar ligands and their applications.

## 4. Materials and Methods

### 4.1. Materials

All solvents used for extractions, chromatography and gelation experiments were fractionally distilled under atmospheric pressure. DMSO (p.a. ACS) was purchased from Sigma Aldrich and was used with no further purification.

### 4.2. General Methods

Analysis with thin layer chromatography (TLC) was made on Silica Gel 60 F254 aluminum-supported plates (layer thickness 0.2 mm) using the indicated solvent systems. Detection of the spots was performed using exposure to UV light and/or charring with Hanessian’s stain. Compounds were purified using flash column chromatography on Silica Gel 60 (230–400 mesh), as described. Optical rotations were determined at 20 °C in a 1 dm cell in a Perkin–Elmer 343 polarimeter with an Na lamp at 589 nm. High-resolution mass spectra (HRMS) were obtained using a Bruker micrOTOF-Q II mass spectrometer with Electrospray Ionization (ESI). ^1^H and ^13^C Nuclear Magnetic Resonance (NMR) spectra were recorded at 25 °C with a Bruker Avance Neo 500 spectrometer at 499.94 and 125.71 MHz, respectively. ^1^H and ^13^C chemical shifts are reported in parts per million relative to tetramethylsilane or a residual solvent peak (CHCl_3_: ^1^H: δ 7.26 ppm, ^13^C: δ 77.2 ppm). Assignment of ^1^H and ^13^C NMR spectra was achieved using ^1^H-COSY, HSQCps and HMBC and 2D ^1^H-^13^C experiments (HSQC-ps, HMBC). Rheology experiments were performed on a Paar Physica MCR 300 rheometer (Anton Paar GmbH, Graz, Austria) with Peltier plate temperature control at 20 °C.

### 4.3. Synthetic Methodologies

#### 4.3.1. 2-(2-Azidoethoxy)ethanol

It was synthesized as previously reported. 2-(2-Cloroetoxy)ethanol (1.18 g, 9.47 mmol) was dissolved in water (6 mL) and NaN_3_ (1.54 g, 23.68 mmol) was added. The mixture was stirred at 80 °C for 18 h, when the TLC showed the transformation of the starting material into the product (R*f* = 0.41, *n*-hexane/EtOAc 1:1). The mixture was poured into 5% NaOH in water (6 mL) and extracted with diethyl ether (4 × 10 mL). The combined organic phase was dried with MgSO_4_, filtered and concentrated. The product was obtained as a colorless oil (1.02 g, 82% yield). ^1^H and ^13^C NMR spectra were coincident with those previously reported [40].

#### 4.3.2. 1-Azido-2-(2-iodoethoxy)ethane

2-(2-Azidoethoxy)ethanol (1.02 g, 7.8 mmol), imidazole (0.69 g, 10.15 mmol), triphenylphosphine (2.66 g, 10.15 mmol) and iodine (2.58 g, 10.15 mmol) were suspended in CH_2_Cl_2_ (50 mL) at 0 °C for 30 min. The mixture was stirred at room temperature for 6 h and then, sodium metabisulfite (sat. sl. 50 mL) was added. The organic phase, after extraction, was dried (MgSO_4_) and the solvent was evaporated. The product (1.43, 76% yield) was purified using column chromatography and eluted with *n*-hexane:EtOAc 9:1 → 8:2. ^1^H and ^13^C NMR spectra were coincident with those previously reported [41].

#### 4.3.3. 2-(2-Azidoethoxy)ethyl 2-acetamido-3,4,5-tri-*O*-acetyl-2-deoxy-1-thio-β-d-glucopyranoside **3**

To a solution of 2-acetamido-3,4,5-tri-*O*-acetyl-2-deoxy-1-thio-β-D-glucopyranoside (200 mg, 0.51 mmol) and thiourea (196 mg, 2.52 mmol) in CH_3_CN (1.1 mL), BF_3_·OEt_2_ (26 µL, 1.50 mmol) was added under Ar. The mixture was refluxed for 3 h, and thiourea (16 mg, 0.21 mmol), together with BF_3_·OEt_2_ (4 µL, 0.21 mmol), was added. The reaction proceeded for 1 h under reflux until the disappearance of the starting peracetylated GlcNAc (R*f* = 0.40, *n*-hexane/EtOAc 1:9) was observed with TLC, which was transformed into thiouronium salt with R*f* = 0.0. The mixture was allowed to reach room temperature and Et_3_N (333 µL, 2.36 mmol) and 1-azido-2-(2-iodoethoxy)ethane (170 mg, 0.70 mmol) were added. After 18 h, TLC showed a new spot of R*f* = 0.45 (*n*-hexane/EtOAc 1:9). The solvent was evaporated, and the residue resuspended in EtOAc and extracted with water. The organic phase was dried (MgSO_4_) and concentrated. Purification of **3** using column chromatography (*n*-hexane:EtOAc 4:6 → 3:7) led first to the pure α anomer (31 mg, 13%). Further elution with *n*-hexane:EtOAc 3:7 → 2:8) led to compound **3** impurified with thiourea. This mixture was dissolved in MeOH/water 3:7 and purified by passing through a C-18 cartridge. Thiourea and salts were eluted with MeOH/water 3:7, while pure compound **3** (127 mg, 52%) was obtained using elution with MeOH/water 1:1. [α${]}_{D}^{20}$ = −18.1 (*c* = 1.0, MeOH); ^1^H NMR [500 MHz, CDCl_3_] δ (ppm) 5.80 (d, 1H, *J*_2,NH_ = 9.5 Hz, CON*H*), 5.14 (t, 1H, *J*_2,3_ ≈ *J*_3,4_ = 9.5 Hz, H-3), 5.09 (t, 1H, *J*_3,4_ ≈ *J*_4,5_ = 9.4 Hz, H-4), 4.75 (d, 1H, *J* = 10.5 Hz, H-1), 4.23 (dd, 1H, *J*_5,6_ = 4.9, *J*_6,6′_ = 12.5 Hz, H-6), 4.13 (dd, 1H, *J*_5,6′_ = 2.4, *J*_6,6′_ = 12.5 Hz, H-6′), 4.09 (ddd, 1H, *J*_1,2_ = 10.5, *J*_2,3_ = *J*_2,NH_ = 9.5 Hz, H-2), 3.77 (m, 1H, C*H*O), 3.69 (m, 1H, H-5), 3.63 (m, 3H, 3C*H*_2_O), 3.45 (m, 2H, C*H*_2_N_3_), 2.99, 2.76 (2m, 1H each, C*H*_2_S), 2.08, 2.02, 2.01, 1.96 (4s, 3H each, 4 × C*H*_3_CO). ^13^C NMR [125.7 MHz, CDCl_3_] δ (ppm) 171.2, 170.9, 170.2, 169.5 (*C*O), 85.1 (*C*1), 76.1 (*C*5), 74.0 (*C*3), 71.9, 69.8 (*C*H_2_O), 68.3 (*C*4), 62.4 (*C*6), 53.5 (*C*2), 51.1 (*C*H_2_N_3_), 29.8 (*C*H_2_S), 23.4, 20.9, 20.8 (2×) (*C*H_3_CO). ESI-HRMS (ESI): *m*/*z* [M+H]^+^ calcd for C_18_H_29_N_4_O_9_S 476.1577; found: 477.1654.

#### 4.3.4. 2-(2-Azidoethoxy)ethyl 2-acetamido-2-deoxy-1-thio-β-D-glucopyranoside **4**

Deacetylation of **3** (166 mg, 0.30 mmol) was carried out by stirring a solution in MeOH/Et_3_N/H_2_O 6:1:3 (vol/vol) for 4 h at room temperature until TLC showed complete transformation into the product (R*f* = 0.56, EtOAc/MeOH/Py 7:3:1). The product was purified by passing through a Dowex MR-3C mixed-bed resin, which was eluted with water. After evaporation, **4** (101 mg, 97% yield) was recovered. [α${]}_{D}^{20}$ = −13.3 (*c* = 1.0, H_2_O); ^1^H NMR [500 MHz, CDCl_3_] δ = 4.70 (d, 1H, *J*_1,2_ = 10.4 Hz, H-1), 3.92 (d, 1H, *J*_6,6′_ = 12.3 Hz, H-6), 3.83–3.71 (m, 6H, H-2, H-6′, 2 × C*H*_2_O), 3.56 (m, 1H, H-3), 3.50 (t, 2H, *J* = 5.1 Hz, C*H*_2_N_3_, 3.48 (m, 2H, H-4, H-5), 3.01, 2.90 (2m, 1H each, *J* = 6.8 Hz, C*H*_2_S), 2.05 (s, 3H, CH_3_CO). ^13^C NMR [125.7 MHz, CD_3_Cl_3_] δ = 174.4 (*C*O), 84.3 (*C*1), 79.9 (*C*5), 75.1 (*C*3), 89.9, 89.8 (*C*H_2_O, *C*4), 89.0 (*C*H_2_O), 60.9 (*C*6), 54.7 (*C*2), 50.2 (*C*H_2_N_3_), 29.4 (*C*H_2_S), 22.1 (*C*H_3_CO). ESI-HRMS (ESI): *m*/*z* [M+H]^+^ calcd for C_12_H_23_N_4_O_6_S 351.1333; found: 351.1330.

#### 4.3.5. General Procedure for the Click Reaction

The reaction was performed as previously described [29]. Compounds **6** or **7** (0.07 mmol) and azide **4** (0.16 mmol, 35 mg) were dissolved in DMF (0.6 mL) and CuI (11 mg) and DIPEA (17 µL) were added. The reaction proceeded for 18 h at room temperature under stirring, until the disappearance of **4** was observed with TLC. The mixture was evaporated and purified using column chromatography using the solvent systems indicated in each case.

Compound **1**. The residue was dissolved in EtOAc/MeOH/Py 50:36:14 and applied to the column which was eluted with EtOAc/MeOH/Py 7:3:1 → 6:4:1. A total of 62 mg were recovered (72%). ^1^H NMR [500MHz, (CD_3_)_2_SO] δ = 8.62 (t, 1H, *J* = 5.7 Hz, CH_2_N*H*), 7.79 (s, 1H, H-triazole), 7.71 (d, 1H, *J*_2,NH_ = 9.4 Hz, AcN*H*), 5.50 (s, 1H, C*H*O), 5.05, 5.02, 4.53 (3 brs, 1H each, 3 O-H), 4.46 (t, 2H, *J* = 5.3 Hz, C*H*_2_Ar), 4.34 (d, 1H, *J*_1,2_ = 10.3 Hz, H-1), 4.31, 4.23 (2 dd, 1H each, *J*_Ha,Hb_ = 15.1, *J*_Ha,NH_ = *J*_Hb,NH_ = 5.8 Hz, Ar-C*H*_2_-NH), 3.76 (t, 2H, *J* = 5.3 Hz, C*H*_2_O), 3.67 (m, 1H, H-6), 3.61–3.38 (m, 4H, C*H*_2_O + H-2 + H-6′), 3.25 (t, 1H, *J*_2,3_ = *J*_3,4_ = 8.6 Hz, H-3), 3.11–3.05 (m, 2H, H-4 + H-5), 2.76, 2.65 (2m, 1H each, *J* = 7.8 Hz, C*H*_2_S);, 2.34;, 2.23 (2m, 1H each, *J* = 7.7 Hz, C*H*_2_CO), 1.78 (s, 3H, C*H*_3_CO), 1.42 (m, 2H, C*H*_2_CH_2_CO);, 1.29–1.20 (m, 8H, 4 × CH_2_), 0.85 (t, 3H, *J* = 6.8 Hz, CH_3_). ^13^C NMR [125.7 MHz, (CD_3_)_2_SO] δ = 172.1, 169.0, 165.8 (3 *C*O), 144.2, 123.2 (*C*-triazole), 84.4 (*C*1), 81.2 (*C*4), 75.5 (*C*3), 72.0 (*C*HO), 70.5 (*C*4), 70.0, 68.4 (*C*H_2_O), 61.2 (*C*6), 54.5 (*C*2), 49.3 (*C*H_2_NTriazole), 34.3 (Ar*C*H_2_NH), 33.2 (*C*H_2_CO), 31.3, (*C*H_2_), 28.7 (*C*H_2_S), 28.4, 28.3, 24.1 (*C*H_2_), 23.0 (*C*H_3_CO), 22.1 (*C*H_2_), 14.0 (*C*H_3_). ESI-HRMS: *m*/*z* [M+H]^+^ calcd for C_50_H_85_N_10_O_18_S_2_ 1177.5480; found: 1177.5492.

Compound **2**. The residue was dissolved in EtOAc/MeOH/Py 75:8:17 and applied to a column which was eluted with EtOAc/MeOH/Py 8:2:1 to recover unreacted **4**. Compound **7** was recovered using elution with EtOAc/MeOH/Py 8:2:1 → 4:6:10. A total of 66 mg were recovered (77%). ^1^H NMR [500 MHz, (CD_3_)_2_SO] δ = ^1^H NMR [500 MHz, (CD_3_)_2_SO] δ = 8.61 (t, 1H, *J* = 5.8 Hz, CH_2_N*H*), 7.79 (s, 1H, H-triazole), 7.71 (d, 1H, *J* = 9.4 Hz, AcN*H*), 5.49 (s, 1H, C*H*O), 5.02, 4.98, 4.52 (3m, 1 H each, 3 O-H), 4.46 (t, 2H, *J* = 5.3 Hz, C*H*_2_Ar), 4.35 (d, 1H, *J*_1,2_ = 10.3 Hz, H-1), 4.33, 4.20 (2 dd, 1 h each, *J*_Ha,Hb_ = 15.2, *J*_Ha,NH_ = *J*_Hb,NH_ = 5.7 Hz, Ar-C*H*_2_-NH), 3.76 (t, 2H, *J* = 5.3 Hz, C*H*_2_O), 3.68 (m, 1H, H-6), 3.62–3.39 (m, 4H, C*H*_2_O + H-2 + H-6′), 3.25 (m, 1H, H-3), 3.12–3.04 (m, 2H, H-4 + H-5), 2.76, 2.64 (2m, 1H each, *J* = 7.6 Hz, C*H*_2_S), 2.37, 2.19 (2m, 1H each, *J* = 7.7 Hz, C*H*_2_CO), 1.79 (s, 3H, CH_3_CO), 1.43 (m, 2H, C*H*_2_CH_2_CO), 1.29–1.20 (m, 18H, 9 × CH_2_), 0.85 (t, 3H, *J* = 6.8 Hz, CH_3_). ^13^C NMR [125.7 MHz, (CD_3_)_2_SO] δ = 172.1, 169.0, 165.9 (3 *C*O), 144.2 (*C*-Triazole), 123.2 (*C*H-Triazole), 84.4 (*C*1), 81.2 (*C*5), 75.5 (*C*3), 72.0 (*C*HO), 70.5 (*C*4), 70.0, 68.4 (*C*H_2_O), 61.2 (*C*6), 54.5 (*C*2), 49.3 (CH_2_ Ntriazole), 34.3 (Ar*C*H_2_NH), 33.2 (*C*H_2_CO), 31.3, 29.1, 29.0, 28.8, 28.9, 28.7 (*C*H_2_), 29.1 (CH_2_S), 24.1 (*C*H_2_), 23.0 (*C*H_3_CO), 22.1 (*C*H_2_), 14.0 (*C*H_3_). ESI-HRMS: *m*/*z* [M+H]^+^ calcd for C_58_H_101_N_10_O_18_S_2_ 1289.6732; found 1289.6731.

Per-acetylated compound **2**: In one case, the insoluble residue obtained after the reaction of azide **4** and scaffold **7** was washed with MeCN, and the remaining material was dissolved in pyridine (0.5 mL) and Ac_2_O (0.5 mL) was added. After 4 h at room temperature, TLC showed a single spot of R*f* = 0.71 (EtOAc/MeOH 7:3). ESI-HRMS: *m*/*z* [M+Na]^+^ calcd for C_70_H_112_N_10_NaO_24_S_2_ 1563.7185; found: 1563.7144.

### 4.4. Characterization

#### 4.4.1. Gelation Assays and Minimal Concentration for Gelation (MCG)

Gelation of compounds **1** and **2** in several solvent systems was tested using 5% *w*/*v* mixtures using the inverted tube method [35]. The mixtures were heated, if insoluble, at room temperature, and then allowed to cool down to room temperature; if the sample could stand under its own weight, it was considered a gel. Sol–gel transition temperatures for gels of compounds **1** and **2** were studied at different concentrations (0.1–5.0% *w*/*v*) in water and DMSO, respectively. The dilutions were heated until complete dissolution and then were allowed to reach room temperature (25 °C). The minimal concentration for gelation (MCG) was the lowest concentration that led to a gel in the solvent used at 25 °C. Sol–gel transition temperatures (T_gel_) were determined using a tube inversion assay with a thermostated water bath.

#### 4.4.2. Scanning Electron Microscopy (SEM)

SEM pictures were taken on a SEM Quanta 250 FEG scanning electron microscope. The sample was attached to the holder using conductive adhesive carbon tape. Prior to examination, they were coated with a thin layer of gold.

#### 4.4.3. Turbidity Assay with Wheat Germ Agglutinin (WGA)

Turbidity experiments were carried out with a UV/Vis Shimatzu UV-2600i spectrometer. The absorbance at 677 nm of a solution of compound **1** (7.07 × 10^−4^ M) or compound **2** (8.67 × 10^−4^ M) in PBS buffer (pH = 7.4, 10 mM) was registered over a period of 4 min. A total of 80 µL of a stock solution of WGA (7.6 × 10^−5^ M) was added at *t* ≈ 3 min. After 35 min, a GlcNAc solution (30 µL, 9.0 × 10^−2^ M) was added. Control experiments under the same conditions but using bovine serum albumin (BSA, 80 µL, 6.0 × 10^−5^ M) were carried out.

#### 4.4.4. Circular Dichroism

CD experiments were performed on a 0.3 wt% hydrogel of compound 1 with a a Jasco J-815 ORDE 402/15 spectropolarimeter with a Jasco PFD-425S/15 temperature controller between 8–80 °C.

#### 4.4.5. Transmission Electron Microscopy (TEM)

TEM pictures were taken with a TEM Philips CM200 microscope (Philips, Eindhoven, The Netherlands). A thin film of the sample was placed on a Formvar/Carbon 300 mesh copper grid by touching the surface of the gels and then the samples were dried under a vacuum.

#### 4.4.6. X-ray Powder Diffraction

X-ray powder diffraction experiments were performed on a Malvern-Panalytical diffractometer, Empyrean model, using a PIXcel3D detector and Cu Kα radiation (1.5406 Å) in a Bragg–Brentano configuration, with a 1/16° incident dispersion slit, a 2θ scan range from 1.7° to 30°, step 0.026° and time per step 600 s. Samples were mounted on a Si low background sample holder. Data were collected with Empyrean Data Collector 3.0C software.

## Figures and Tables

**Table 1:** Solubility and gelation scope of amphiphiles **1** and **2** (inverted tube test) and minimal concentration for gelation (MCG).

	Compound 1		Compound 2	
Solvent	Sol/Gel ^1^	MCG (wt%)	Sol/Gel ^1^	MCG (wt%)
Water	G	0.3	P	-
Chloroform	I	-	I	-
Dichloromethane	I	-	I	-
Acetone	I	-	I	-
Ethanol	P	-	P	-
Ethyl acetate	I	-	I	-
*n*-Hexane	I	-	I	-
Ethanol:water 2:1	S	-	P	-
Methanol:water 2:1	S	-	P	-
DMSO	S	-	G	0.8
Methanol	P	-	S	-

^1^ G—gel; S—soluble at room temperature; P—soluble after heating but precipitates when cooled down to room temperature; I—insoluble even after heating.

## Data Availability

Data are contained within the article and Supplementary Materials.

## Supplementary Materials

The following supporting information can be downloaded at: https://www.mdpi.com/article/10.3390/gels10010005/s1, Copies of ^1^H and ^13^C NMR spectra of all amphiphiles, SEM images of xerogels, rheology and T vs. concentration plots. Figure S1: ^1^H and ^13^C NMR spectra (500 and 125 MHz respectively). Figure S2: T_gel_ vs. concentration plot for hydrogels of **1**. Figure S3: T_gel_ vs. concentration plot for DMSO gels of **2**. Figure S4: Reology. Dynamic strain sweep experiment of a hydrogel of **1** an DMSO gel of **2** at 25 °C. Figure S5: Powder XRD experiments. Figure S6: SEM images obtained from xerogels of a hydrogel of **1** (a) and a DMSO gel of **2** (b). Figure S7: TEM image of the xerogel obtained from **2**.

## References

[1] Sorrenti A., Illa O., Ortuño R.M. (2013). Amphiphiles in Aqueous Solution: Well beyond a Soap Bubble. Chem. Soc. Rev..

[2] Argudo P.G., Spitzer L., Jerome F., Cramail H., Camacho L., Lecommandoux S. (2022). Design and Self-Assembly of Sugar-Based Amphiphiles: Spherical to Cylindrical Micelles. Langmuir.

[3] Ogawa S. (2023). Aqueous Sugar-Based Amphiphile Systems: Recent Advances in Phase Behavior and Nanoarchitectonics. J. Oleo Sci..

[4] Varki A., Cummings R.D., Esko J.D., Stanley P., Hart G.W., Aebi M., Mohnen D., Kinoshita T., Packer N.H., Prestegard J.H. (2017). Essentials of Glycobiology.

[5] Ambrosi M., Cameron N.R., Davis B.G. (2005). Lectins: Tools for the Molecular Understanding of the Glycocode. Org. Biomol. Chem..

[6] Gupta A., Gupta G.S. (2022). Applications of Mannose-Binding Lectins and Mannan Glycoconjugates in Nanomedicine. J. Nanopart. Res..

[7] Ye Z., Zhang Q., Wang S., Bharate P., Varela-Aramburu S., Lu M., Seeberger P.H., Yin J. (2016). Tumour-Targeted Drug Delivery with Mannose-Functionalized Nanoparticles Self-Assembled from Amphiphilic Β-Cyclodextrins. Chem. Eur. J..

[8] Vijayan M., Chandra N. (1999). Lectins. Curr. Opin. Struct. Biol..

[9] Katoch R., Tripathi A. (2021). Research Advances and Prospects of Legume Lectins. J. Biosci..

[10] Barre A., Van Damme E.J.M., Klonjkowski B., Simplicien M., Sudor J., Benoist H., Rougé P. (2022). Legume Lectins with Different Specificities as Potential Glycan Probes for Pathogenic Enveloped Viruses. Cells.

[11] Cavada B.S., de Oliveira M.V., Osterne V.J.S., Pinto-Junior V.R., Martins F.W.V., Correia-Neto C., Pinheiro R.F., Leal R.B., Nascimento K.S. (2023). Recent Advances in the Use of Legume Lectins for the Diagnosis and Treatment of Breast Cancer. Biochimie.

[12] Balčiūnaitė-Murzienė G., Dzikaras M. (2021). Wheat Germ Agglutinin—From Toxicity to Biomedical Applications. Appl. Sci..

[13] Goyard D., Ortiz A.M.-S., Boturyn D., Renaudet O. (2022). Multivalent Glycocyclopeptides: Conjugation Methods and Biological Applications. Chem. Soc. Rev..

[14] Schwefel D., Maierhofer C., Beck J.G., Seeberger S., Diederichs K., Möller H.M., Welte W., Wittmann V. (2010). Structural Basis of Multivalent Binding to Wheat Germ Agglutinin. J. Am. Chem. Soc..

[15] Sharon N., Lis H. (2007). Lectins.

[16] Auth J., Fröba M., Große M., Rauch P., Ruetalo N., Schindler M., Morokutti-Kurz M., Graf P., Dolischka A., Prieschl-Grassauer E. (2021). Lectin from Triticum Vulgaris (WGA) Inhibits Infection with SARS-CoV-2 and Its Variants of Concern Alpha and Beta. Int. J. Mol. Sci..

[17] Pellegrina C.D., Perbellini O., Scupoli M.T., Tomelleri C., Zanetti C., Zoccatelli G., Fusi M., Peruffo A., Rizzi C., Chignola R. (2009). Effects of Wheat Germ Agglutinin on Human Gastrointestinal Epithelium: Insights from an Experimental Model of Immune/Epithelial Cell Interaction. Toxicol. Appl. Pharmacol..

[18] Van Buul V.J., Brouns F.J.P.H. (2014). Health Effects of Wheat Lectins: A Review. J. Cereal Sci..

[19] Montenegro H.O., Di Chenna P.H., Spagnuolo C.C., Uhrig M.L. (2019). Multivalent Assembly of a Pyrene Functionalized Thio-N-Acetylglucosamine: Synthesis, Spectroscopic and WGA Binding Studies. Carbohydr. Res..

[20] Morris J., Bietsch J., Bashaw K., Wang G. (2021). Recently Developed Carbohydrate Based Gelators and Their Applications. Gels.

[21] Rosa Nunes D., Reche-Tamayo M., Ressouche E., Raynal M., Isare B., Foury-Leylekian P., Albouy P.-A., Brocorens P., Lazzaroni R., Bouteiller L. (2019). Organogel Formation Rationalized by Hansen Solubility Parameters: Shift of the Gelation Sphere with the Gelator Structure. Langmuir.

[22] Liu M., Ouyang G., Niu D., Sang Y. (2018). Supramolecular Gelatons: Towards the Design of Molecular Gels. Org. Chem. Front..

[23] Dastidar P. (2019). Designing Supramolecular Gelators: Challenges, Frustrations, and Hopes. Gels.

[24] Prathap A., Sureshan K.M. (2019). Sugar-Based Organogelators for Various Applications. Langmuir.

[25] Dastidar P., Roy R., Parveen R., Sarkar K. (2019). Supramolecular Synthon Approach in Designing Molecular Gels for Advanced Therapeutics. Adv. Ther..

[26] Mukherjee A. (2015). Building upon Supramolecular Synthons: Some Aspects of Crystal Engineering. Cryst. Growth Des..

[27] Mittal A., Krishna, Aarti, Prasad S., Mishra P.K., Sharma S.K., Parshad B. (2021). Self-Assembly of Carbohydrate-Based Small Amphiphiles and Their Applications in Pathogen Inhibition and Drug Delivery: A Review. Mater. Adv..

[28] Faig A., Arthur T.D., Fitzgerald P.O., Chikindas M., Mintzer E., Uhrich K.E. (2015). Biscationic Tartaric Acid-Based Amphiphiles: Charge Location Impacts Antimicrobial Activity. Langmuir.

[29] Cano M.E., Di Chenna P.H., Lesur D., Wolosiuk A., Kovensky J., Uhrig M.L. (2017). Chirality Inversion, Supramolecular Hydrogelation and Lectin Binding of Two Thiolactose Amphiphiles Constructed on a Di-Lauroyl-L-Tartaric Acid Scaffold. New J. Chem..

[30] Almeida M.M., Perez K.R., Faig A., Uhrich K.E., Riske K.A. (2019). Location of the Positive Charges in Cationic Amphiphiles Modulates Their Mechanism of Action against Model Membranes. Langmuir.

[31] Shankar B.V., Patnaik A. (2007). A New PH and Thermo-Responsive Chiral Hydrogel for Stimulated Release. J. Phys. Chem. B.

[32] Novotný M., Hrabálek A., Janůšová B., Novotný J., Vávrová K. (2009). Dicarboxylic Acid Esters as Transdermal Permeation Enhancers: Effects of Chain Number and Geometric Isomers. Bioorg. Med. Chem. Lett..

[33] Raghavan V., Polavarapu P.L. (2017). Specific Optical Rotation Is a Versatile Tool for the Identification of Critical Micelle Concentration and Micellar Growth of Tartaric Acid–Based Diastereomeric Amphiphiles. Chirality.

[34] Kosma P., Wrodnigg T., Stütz A. (2021). Carbohydrate Chemistry.

[35] Weiss R.G., Weiss R.G., Terech P. (2006). Molecular Gels: Materials with Self-Assembled Fibrillar Networks.

[36] Dawn A., Kumari H. (2018). Low Molecular Weight Supramolecular Gels Under Shear: Rheology as the Tool for Elucidating Structure-Function Correlation. Chem. Eur. J..

[37] Lundquist J.J., Toone E.J. (2002). The Cluster Glycoside Effect. Chem. Rev..

[38] Wang K.-R., An H.-W., Wang Y.-Q., Zhang J.-C., Li X.-L. (2013). Multivalent Glycoclusters Constructed by Chiral Self-Assembly of Mannose Functionalized Perylene Bisimide. Org. Biomol. Chem..

[39] Cairo C.W., Gestwicki J.E., Kanai M., Kiessling L.L. (2002). Control of Multivalent Interactions by Binding Epitope Density. J. Am. Chem. Soc..

[40] Gan W., Cao X., Shi Y., Gao H. (2020). Chain-growth Polymerization of Azide–Alkyne Difunctional Monomer: Synthesis of Star Polymer with Linear Polytriazole Arms from a Core. J. Polym. Sci..

[41] Cristófalo A.E., Cagnoni A.J., Uhrig M.L. (2020). Synthesis of *N*-Acetylglucosamine and *N*-Acetylallosamine Resorcinarene-Based Multivalent β-Thio-Glycoclusters: Unexpected Affinity of *N*-Acetylallosamine Ligands towards Wheat Germ Agglutinin. Org. Biomol. Chem..

