# Supplementary material for: Amphiphilic Low-Molecular-Weight Gelators Bearing β-S-N-Acetylglucosamine Linked to a Tartaric Acid Scaffold: Synthesis, Self-Assembly and Wheat Germ Agglutinin Binding
Source: Gels. 2023 Dec 21;10(1):5. doi: 10.3390/gels10010005 (PMC10815405; doi:10.3390/gels10010005)

Supplementary Materials

Amphiphilic Low-Molecular-Weight Gelators Bearing β-S-N-Acetylglucosamine Linked to a Tartaric Acid Scaffold: Synthesis, Self-Assembly and Wheat Germ Agglutinin Binding

Vicente Leafar Peña García[1,2,3], Pablo Héctor Di Chenna[1,3,*] and Maria Laura Uhrig[1,2,*]

1 Departamento de Química Orgánica, Facultad de Ciencias Exactas y Naturales, Universidad de Buenos Aires, Intendente Güiraldes 2160, 3er piso, Pabellón 2, Ciudad Universitaria, Buenos Aires C1428EGA, Argentina; vicentepena@qo.fcen.uba.ar
2 Centro de Investigaciones en Hidratos de Carbono (CIHIDECAR), Consejo Nacional de Investigaciones Científicas y Técnicas (CONICET)–Universidad de Buenos Aires, Buenos Aires C1428EGA, Argentina
3 Unidad de Microanálisis y Métodos Físicos en Química Orgánica (UMYMFOR), Consejo Nacional de Investigaciones Científicas y Técnicas (CONICET)–Universidad de Buenos Aires, Buenos Aires C1428EGA, Argentina
* Correspondence: dichenna@qo.fcen.uba.ar (P.H.D.C.); mluhrig@qo.fcen.uba.ar (M.L.U.)

Figure S1. ^{1}H and ^{13}C NMR spectra (500 and 125 MHz respectively).

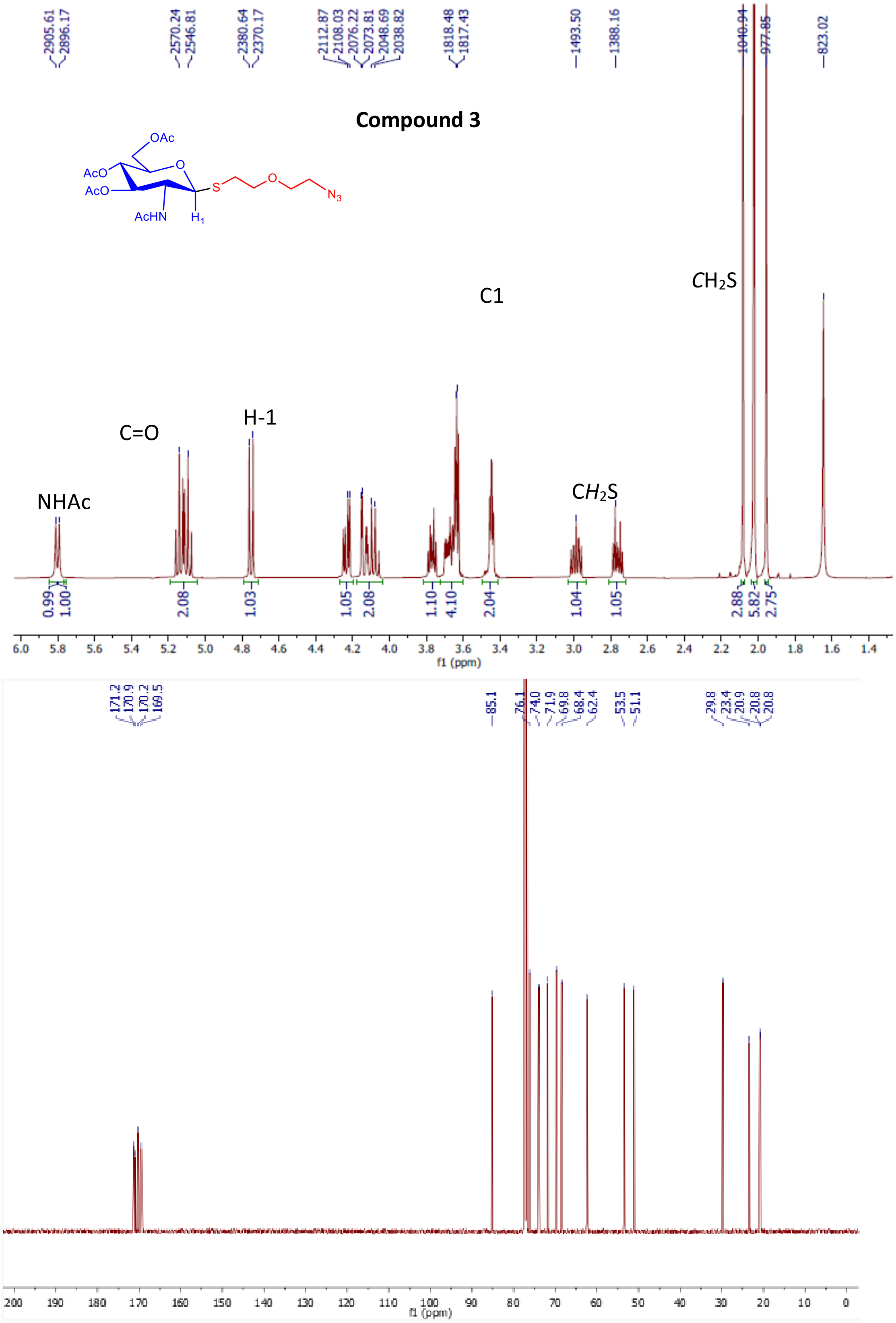

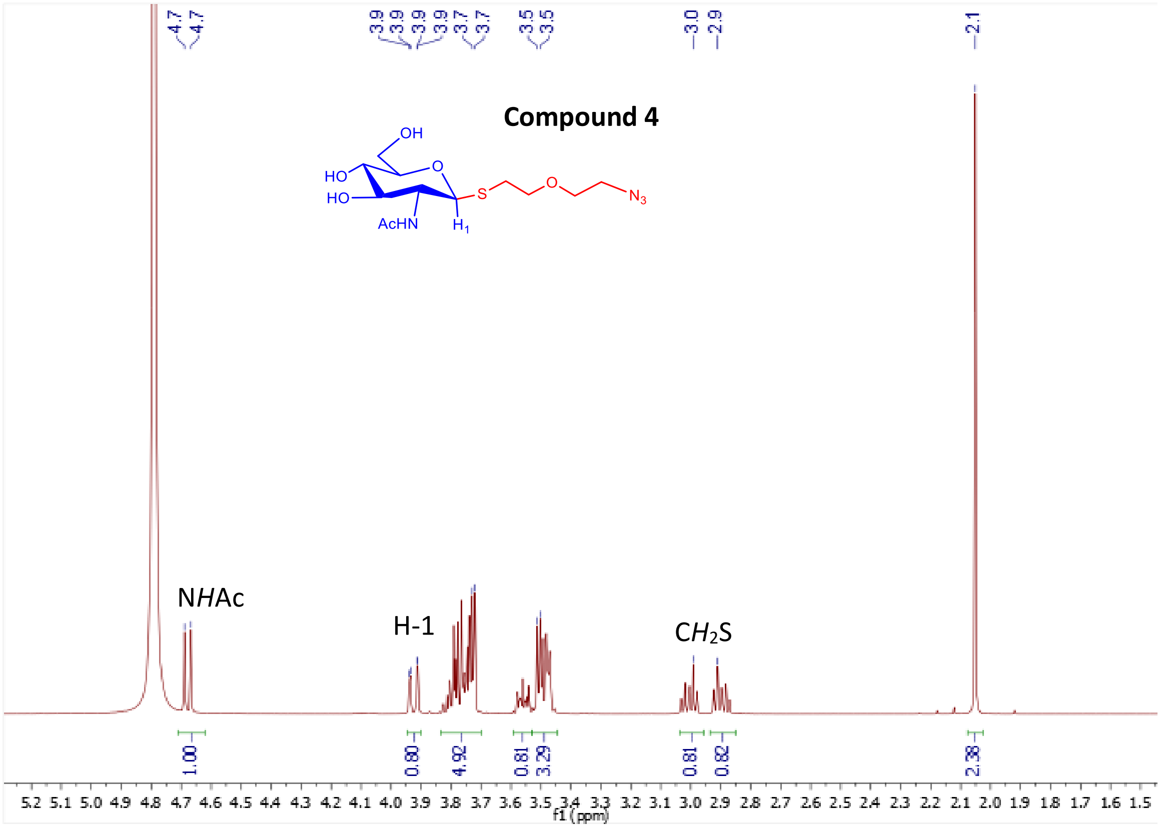

4.7
4.7
3.9
3.9
3.9
3.9
3.7
3.7
3.5
3.5
3.0
2.9
2.1
Compound 4
OH
O
HO
HO
S
O
N3
AcHN
H1
NHAc
H-1
CH2S
1.00
0.80
4.92
0.81
3.29
0.81
0.82
2.38
5.2 5.1 5.0 4.9 4.8 4.7 4.6 4.5 4.4 4.3 4.2 4.1 4.0 3.9 3.8 3.7 3.6 3.5 3.4 3.3 3.2 3.1 3.0 2.9 2.8 2.7 2.6 2.5 2.4 2.3 2.2 2.1 2.0 1.9 1.8 1.7 1.6 1.5
f1 (ppm)

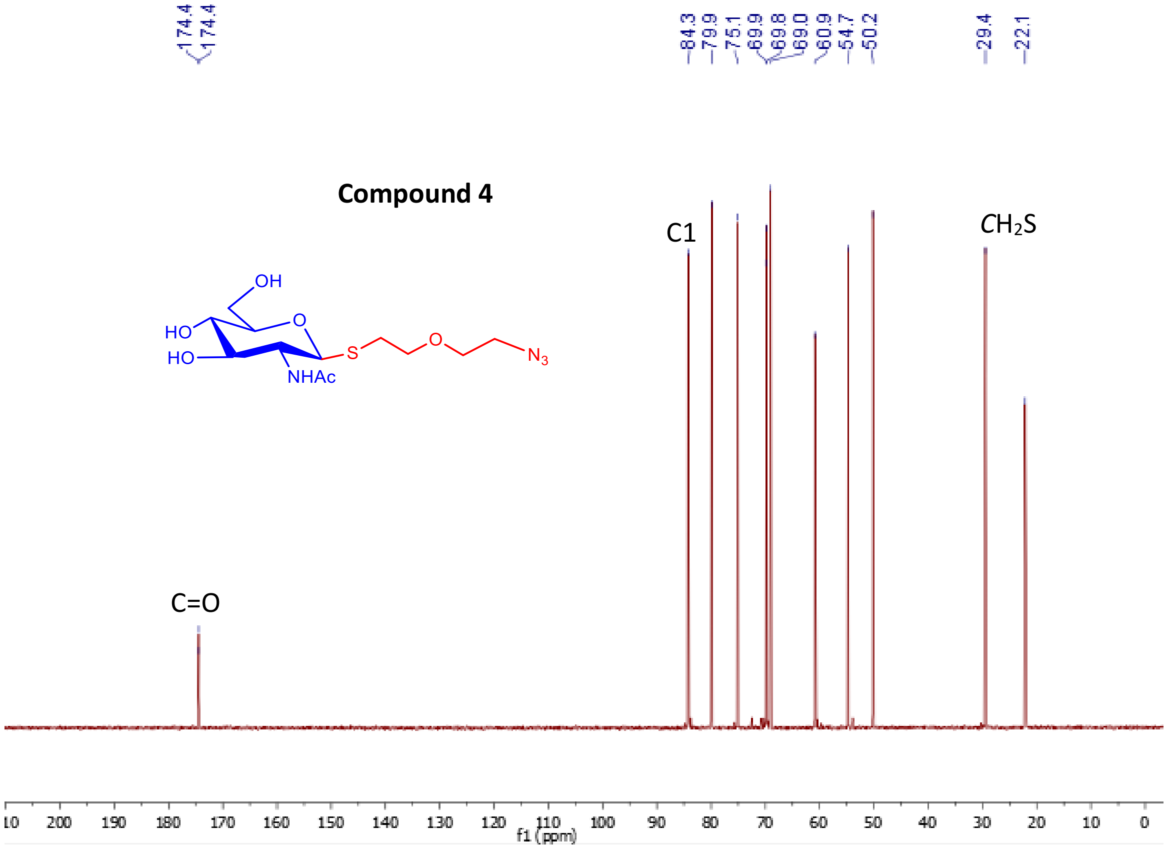

174.4
174.4
84.3
79.9
75.1
69.9
69.8
69.0
60.9
54.7
50.2
29.4
22.1
Compound 4
OH
O
HO
HO
S
O
N3
NHAc
C1
CH2S
C=O
10 200 190 180 170 160 150 140 130 120 110 100 90 80 70 60 50 40 30 20 10 0
f1 (ppm)

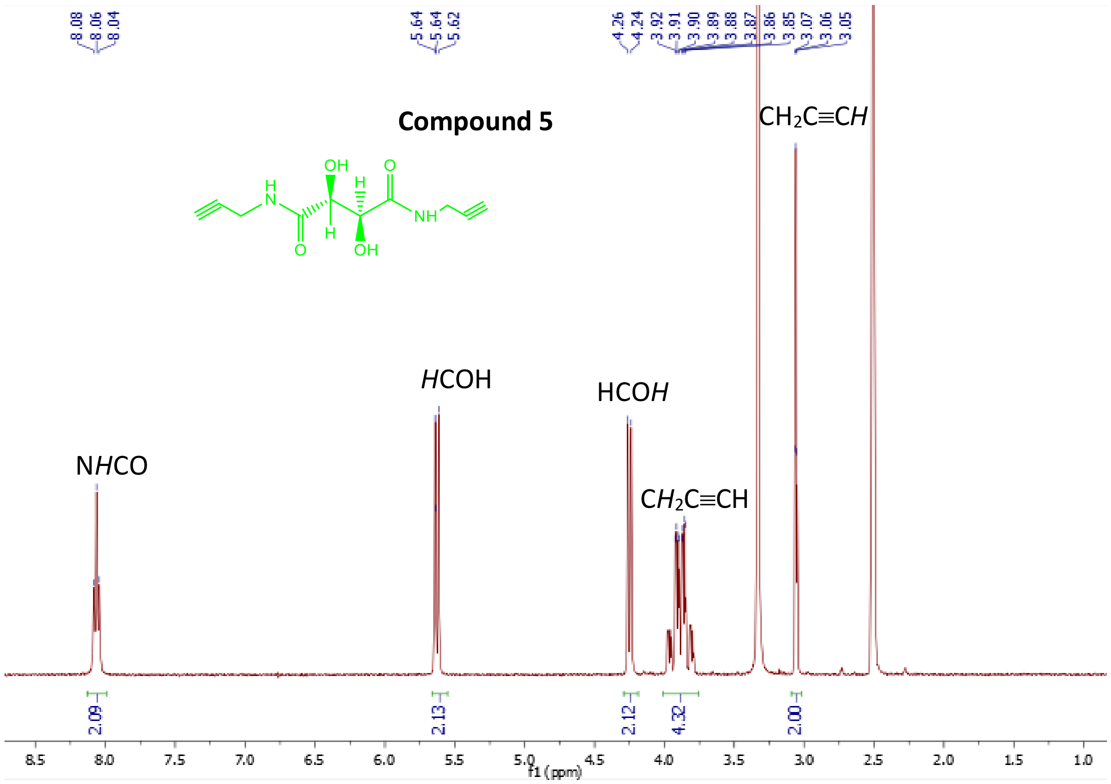

8.08
8.06
8.04
5.64
5.64
5.62
4.26
4.24
3.92
3.91
3.90
3.89
3.88
3.87
3.86
3.85
3.07
3.06
3.05
Compound 5
CH2C≡CH
HCOH
HCOH
NHCO
CH2C≡CH
2.09
2.13
2.12
4.32
2.00
8.5
8.0
7.5
7.0
6.5
6.0
5.5
5.0
4.5
4.0
3.5
3.0
2.5
2.0
1.5
1.0
f1 (ppm)

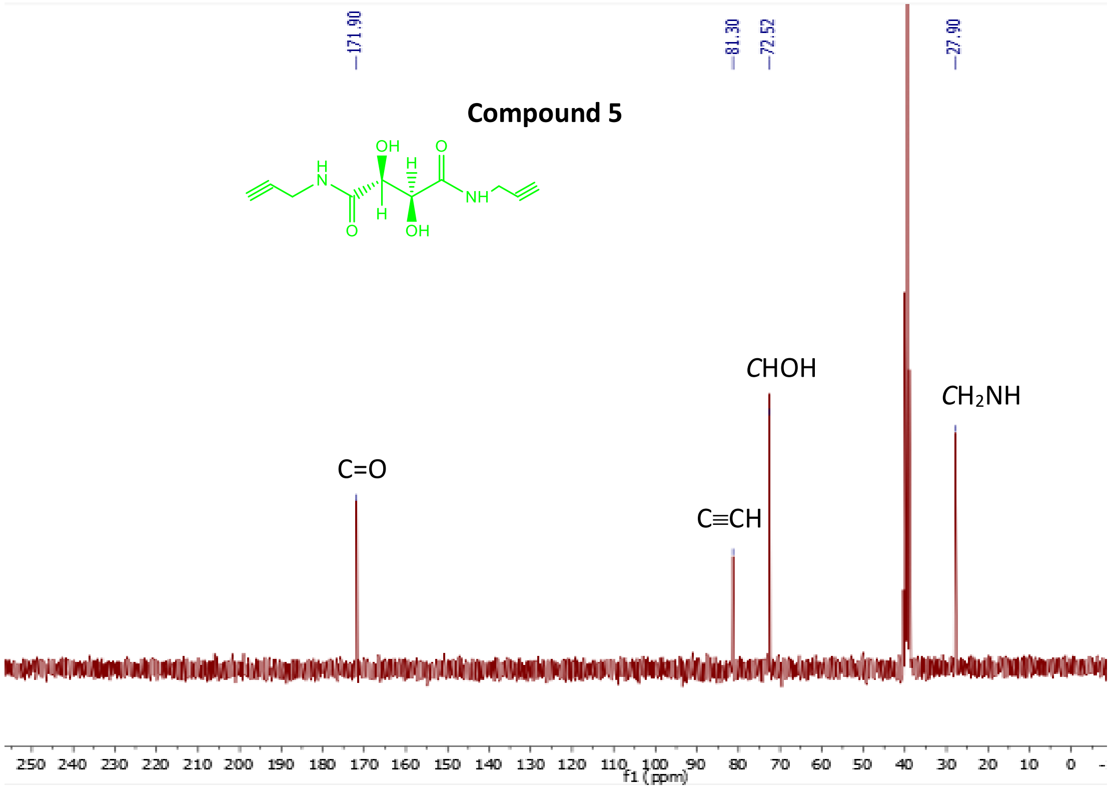

171.90
81.30
72.52
27.90
Compound 5
CHOH
CH2NH
C=O
C≡CH
250 240 230 220 210 200 190 180 170 160 150 140 130 120 110 100 90 80 70 60 50 40 30 20 10 0
f1 (ppm)

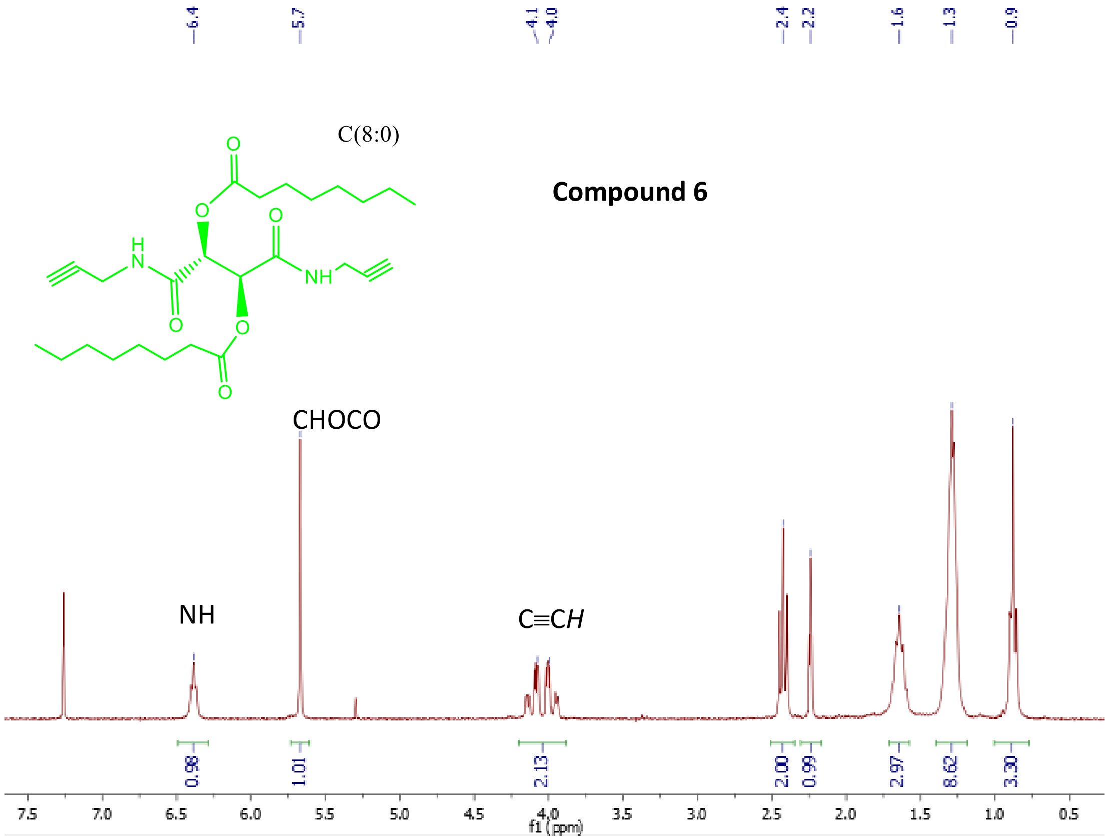
6.4
5.7
4.1
4.0
2.4
2.2
1.6
1.3
0.9
C(8:0)
Compound 6
CHOCO
NH
C≡CH
0.98
1.01
2.13
2.00
0.99
2.97
8.62
3.30
7.5 7.0 6.5 6.0 5.5 5.0 4.5 4.0 3.5 3.0 2.5 2.0 1.5 1.0 0.5
f1 (ppm)

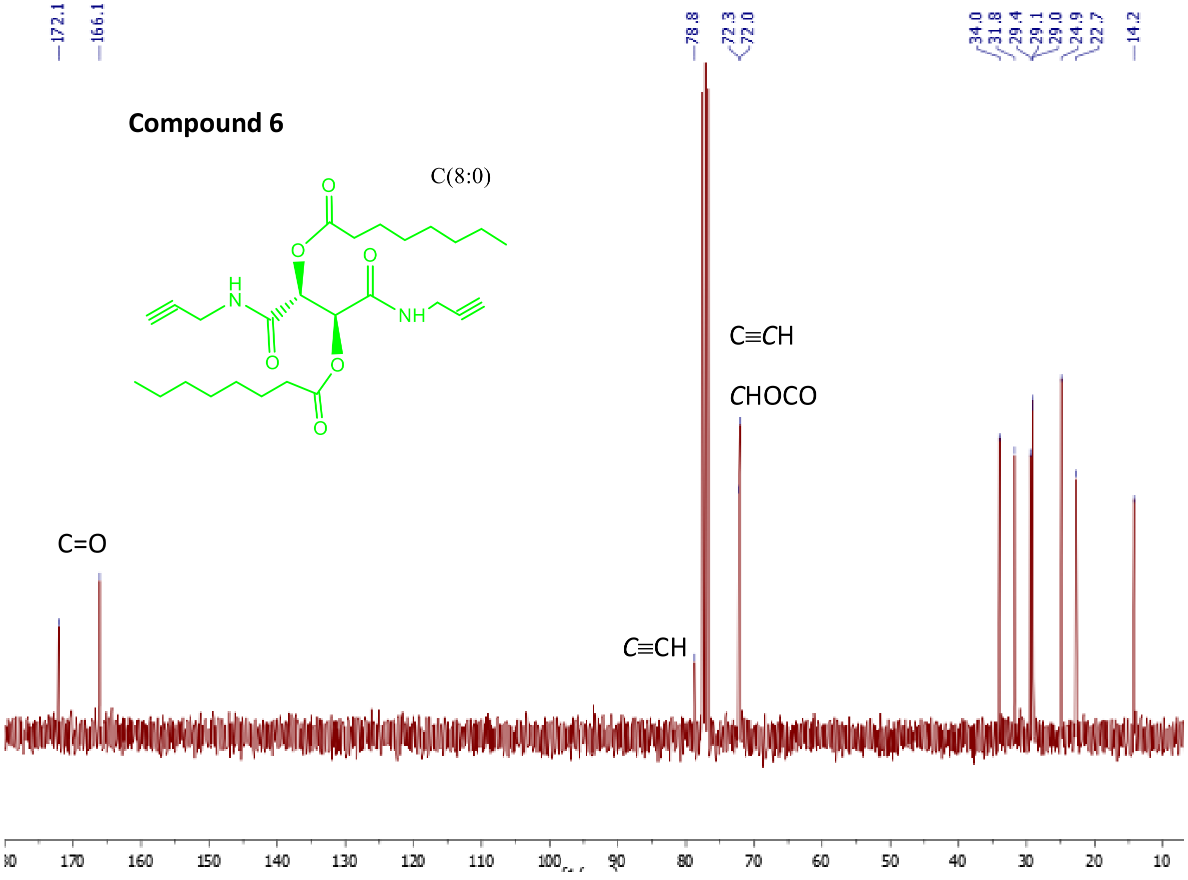
172.1
166.1
78.8
72.3
72.0
34.0
31.8
29.4
29.1
29.0
24.9
22.7
14.2
Compound 6
C(8:0)
C≡CH
CHOCO
C=O
C≡CH
170 160 150 140 130 120 110 100 90 80 70 60 50 40 30 20 10

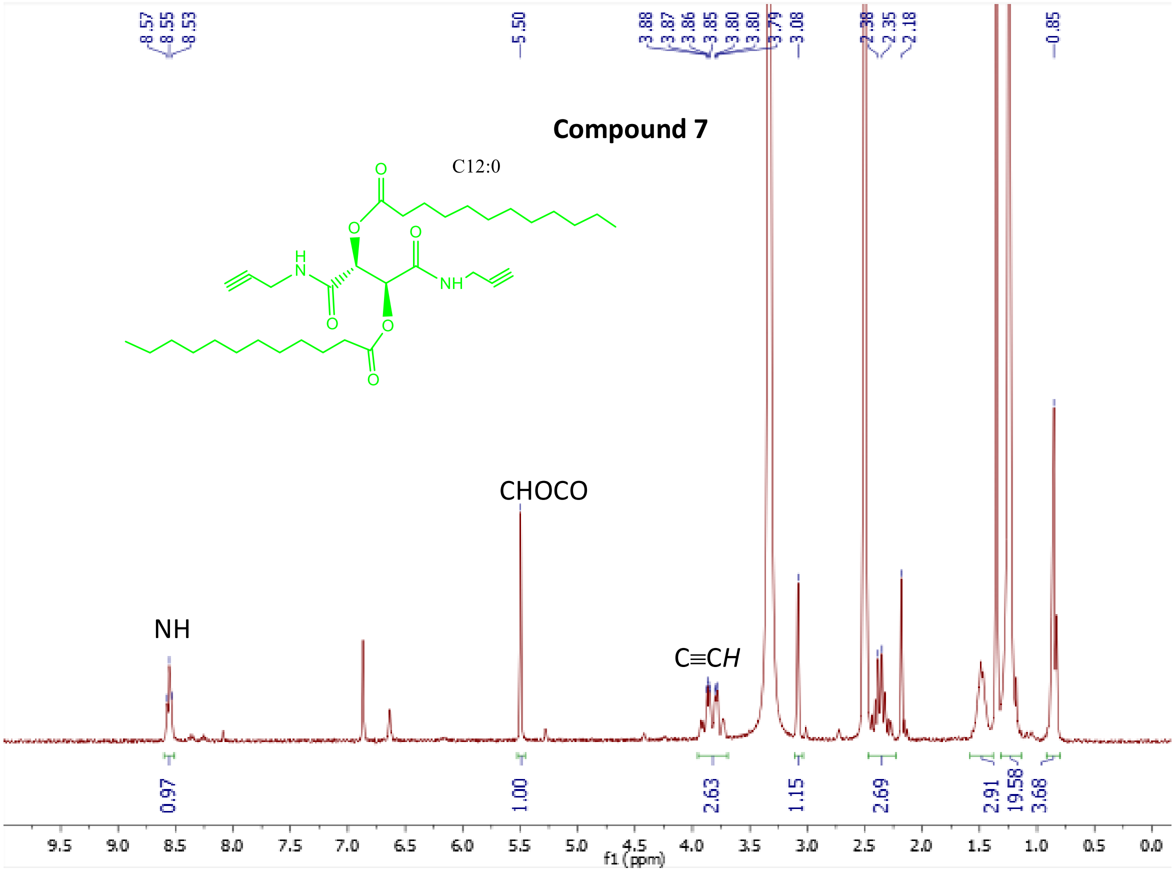
Compound 7
C12:0
CHOCO
NH
C≡CH
f1 (ppm)

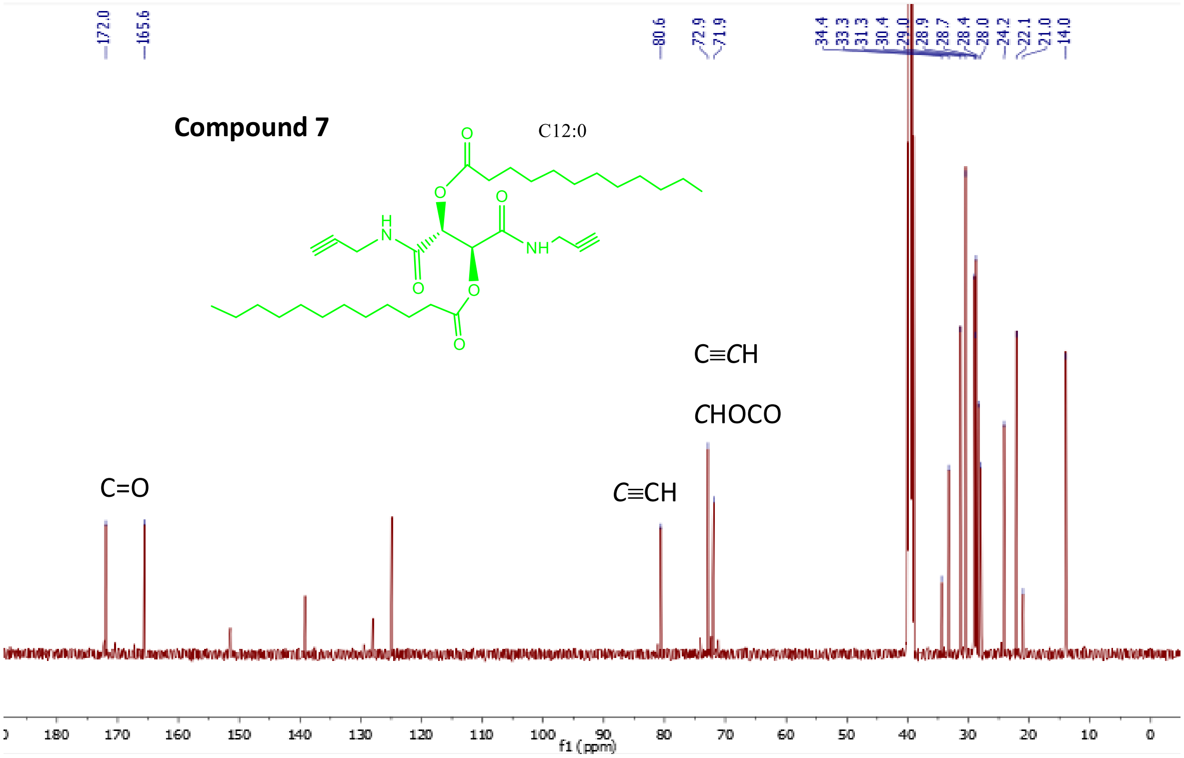
Compound 7
C12:0
C≡CH
CHOCO
C=O
C≡CH
f1 (ppm)

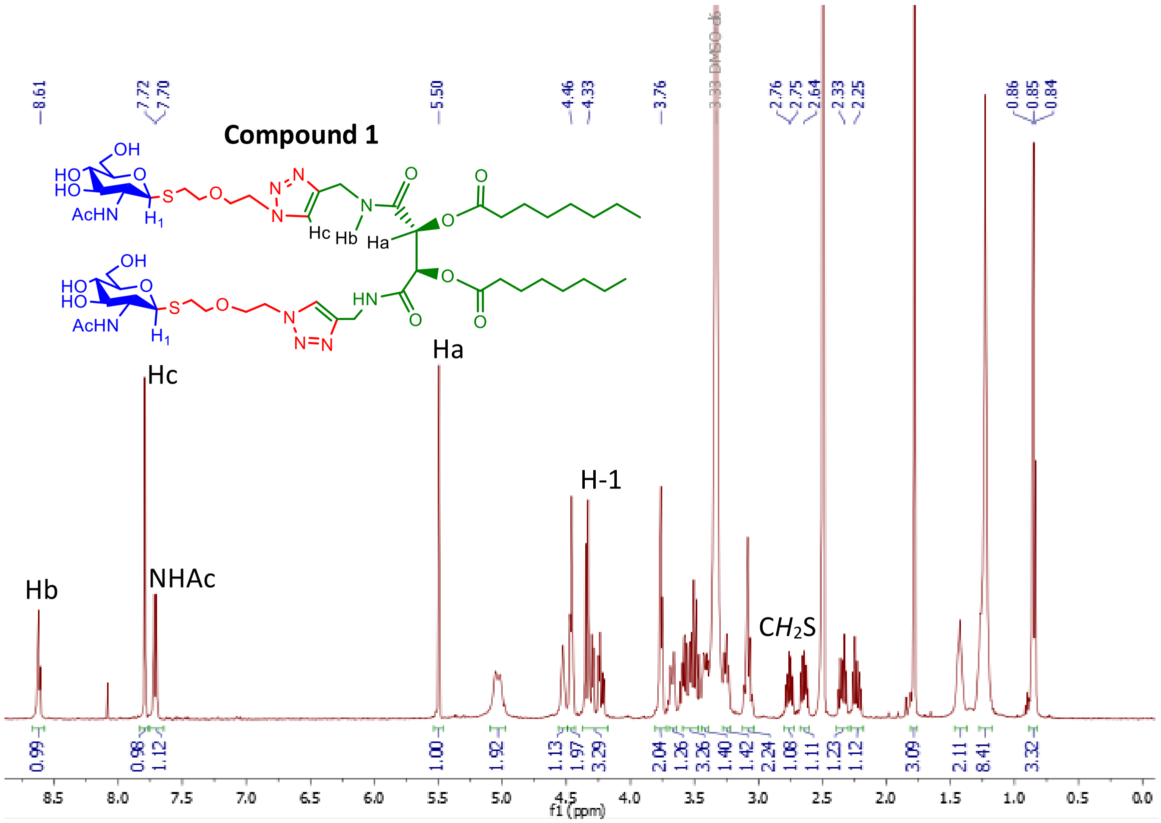
Compound 1
8.61
7.72
7.70
5.50
4.46
4.33
3.76
3.33 DMSO-d6
2.76
2.75
2.64
2.33
2.25
0.86
0.85
0.84
Hb
Hc
NHAc
Ha
H-1
CH_2S
0.99
0.98
1.12
1.00
1.92
1.13
1.97
3.29
2.04
1.26
3.26
1.40
1.42
2.24
1.08
1.11
1.23
1.12
3.09
2.11
8.41
3.32
f1 (ppm)

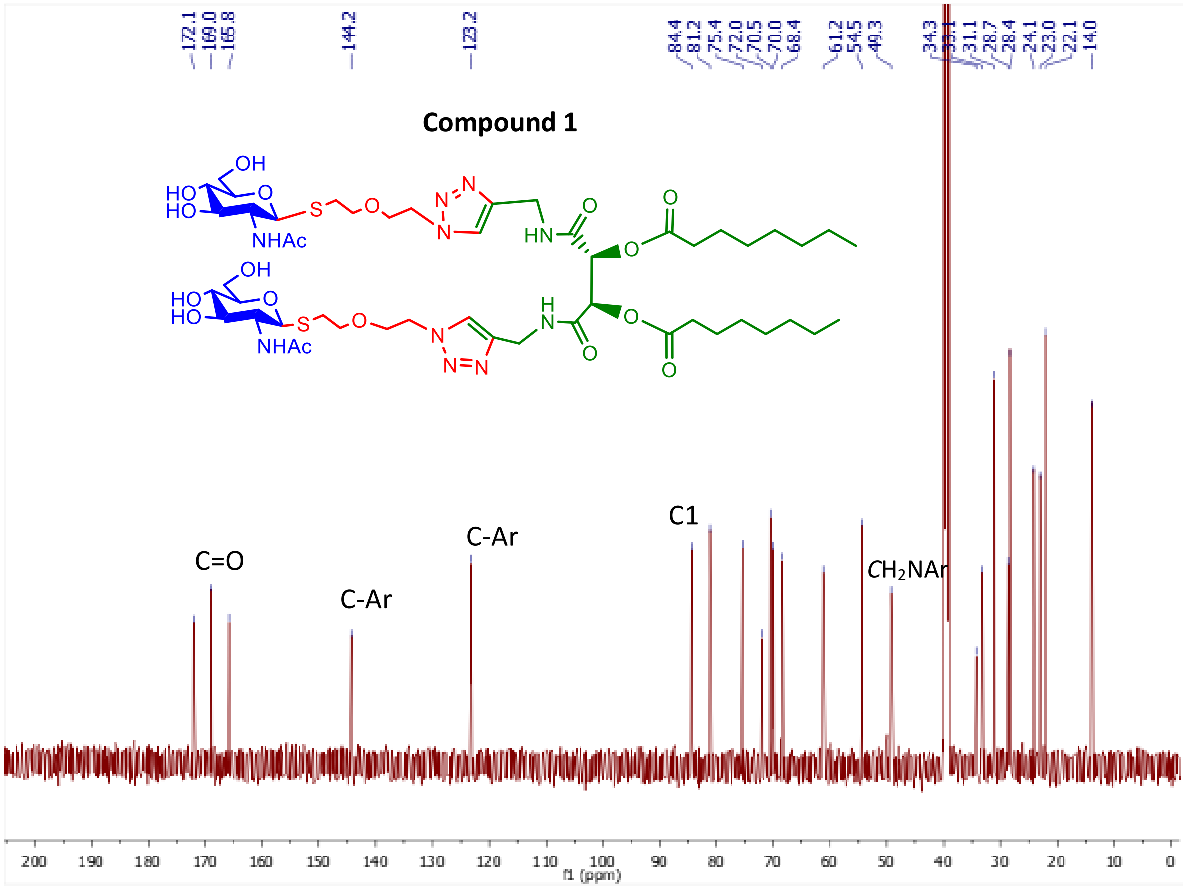
Compound 1
172.1
169.0
165.8
144.2
123.2
84.4
81.2
75.4
72.0
70.5
70.0
68.4
61.2
54.5
49.3
34.3
33.1
31.1
28.7
28.4
24.1
23.0
22.1
14.0
C=O
C-Ar
C-Ar
C1
CH_2NAr
f1 (ppm)

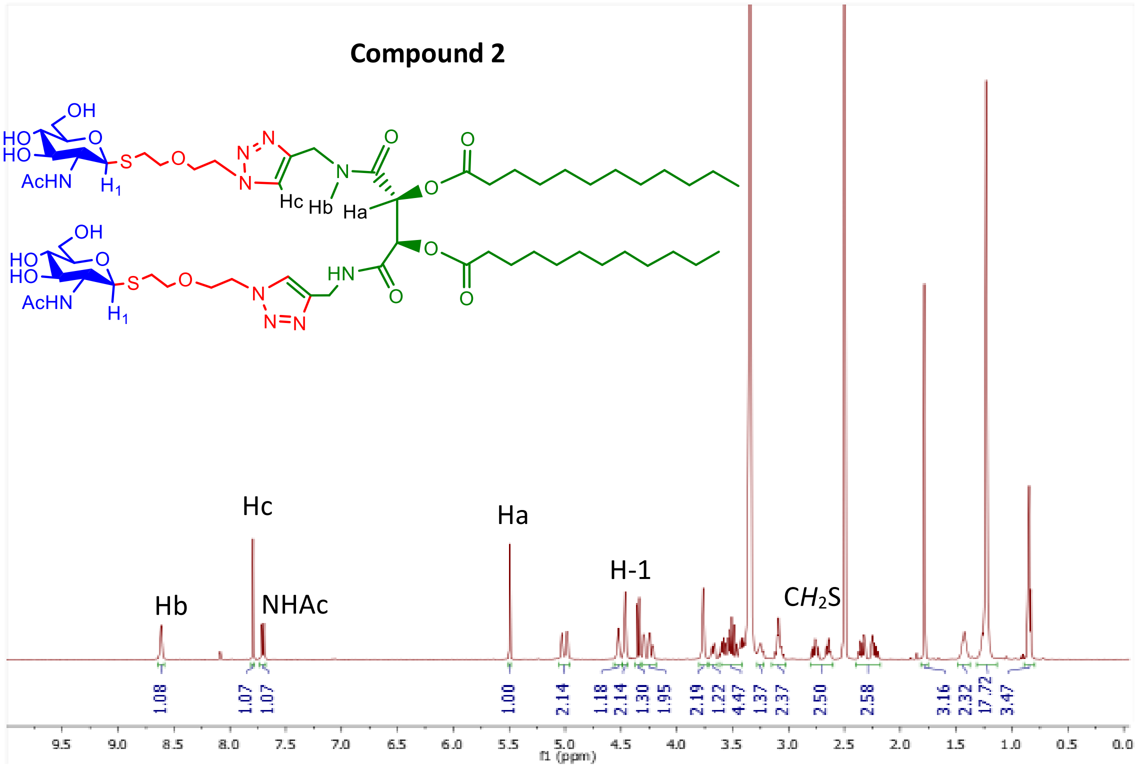
Compound 2
Hb
Hc
NHAc
Ha
H-1
CH2S
1.08
1.07
1.07
1.00
2.14
1.18
2.14
1.30
1.95
2.19
1.22
4.47
1.37
2.37
2.50
2.58
3.16
2.32
17.72
3.47
9.5 9.0 8.5 8.0 7.5 7.0 6.5 6.0 5.5 5.0 4.5 4.0 3.5 3.0 2.5 2.0 1.5 1.0 0.5 0.0
f1 (ppm)

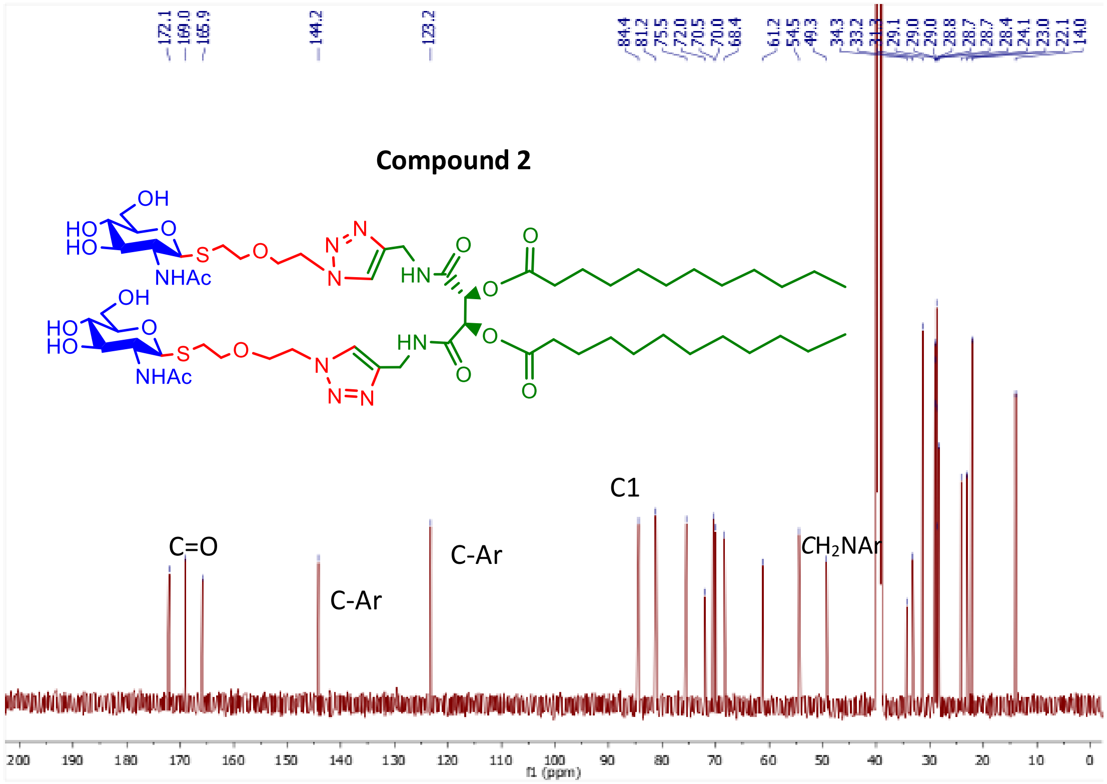
172.1
169.0
165.9
144.2
123.2
84.4
81.2
75.5
72.0
70.5
70.0
68.4
61.2
54.5
49.3
34.3
33.2
31.3
29.1
29.0
29.0
28.8
28.7
28.7
28.4
24.1
23.0
22.1
14.0
Compound 2
C=O
C-Ar
C-Ar
C1
CH2NAr
200 190 180 170 160 150 140 130 120 110 100 90 80 70 60 50 40 30 20 10 0
f1 (ppm)

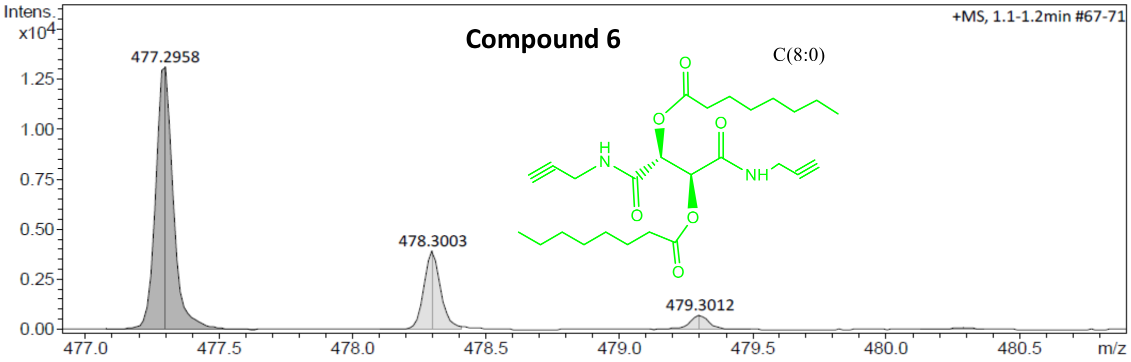

Meas. m/z	#	Ion Formula	m/z	err [ppm]	mSigma	# mSigma	Score	rdb	e⁻ Conf	N-Rule
477.2958	1	C26H41N2O6	477.2959	0.2	4.1	1	100.00	7.5	even	ok

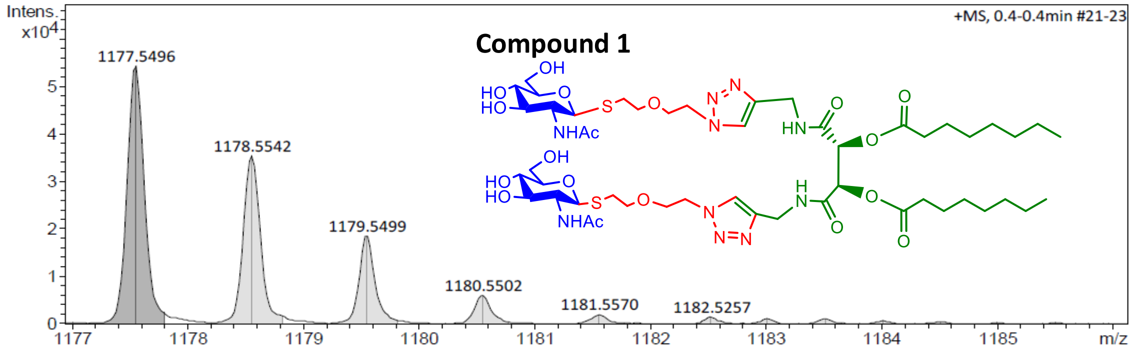

Meas. m/z	#	Ion Formula	m/z	err [ppm]	mSigma	# mSigma	Score	rdb	e⁻ Conf	N-Rule
1177.5496	1	C50H85N10O18S2	1177.5479	-1.4	22.1	1	100.00	13.5	even	ok

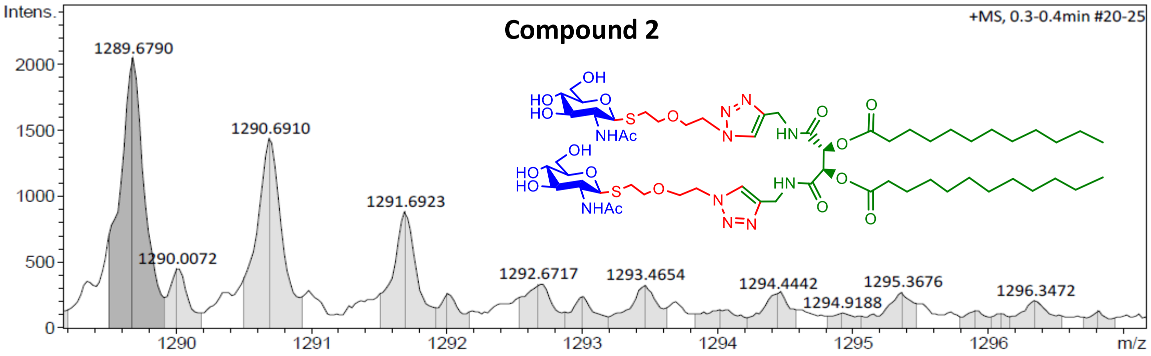

Meas. m/z	#	Ion Formula	m/z	err [ppm]	mSigma	# mSigma	Score	rdb	e⁻ Conf	N-Rule
1289.6790	1	C58H101N10O18S2	1289.6731	-4.6	34.1	1	20.56	13.5	even	ok

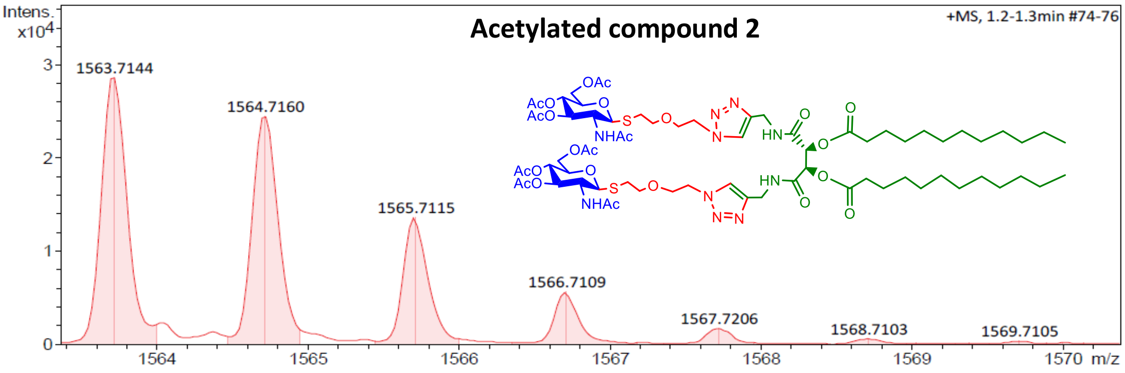

Meas. m/z	#	Ion Formula	m/z	err [ppm]	mSigma	# mSigma	Score	rdb	e¯ Conf	N-Rule
1563.7144	1	C70H112N10NaO24S2	1563.7185	2.6	11.8	1	100.00	19.5	even	ok

Figure S2. T_{gel} *vs* concentration plot for hydrogels of **1**

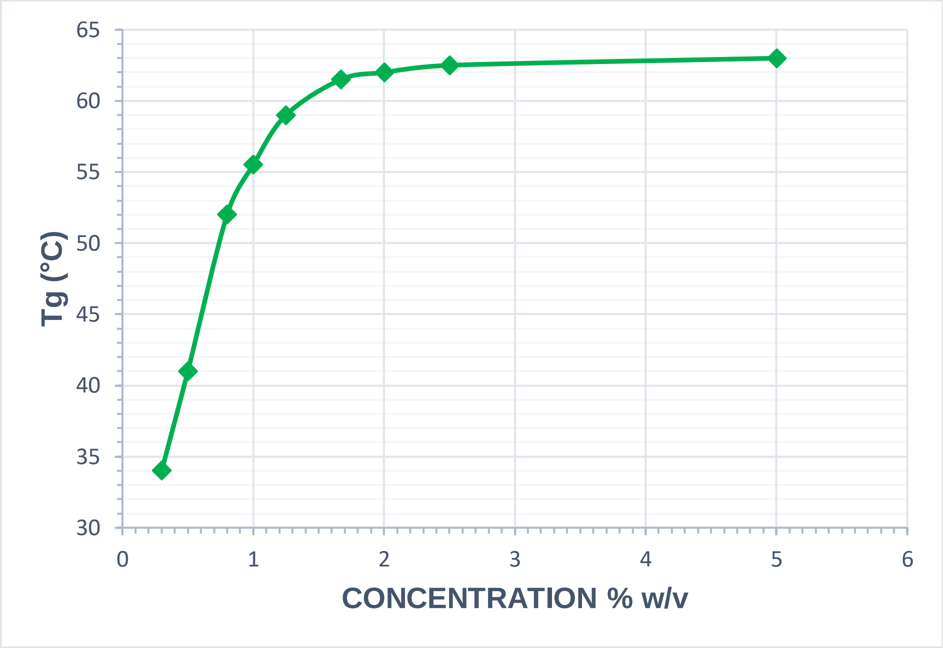

Figure S3. T_{gel} *vs* concentration plot for DMSO gels of **2**

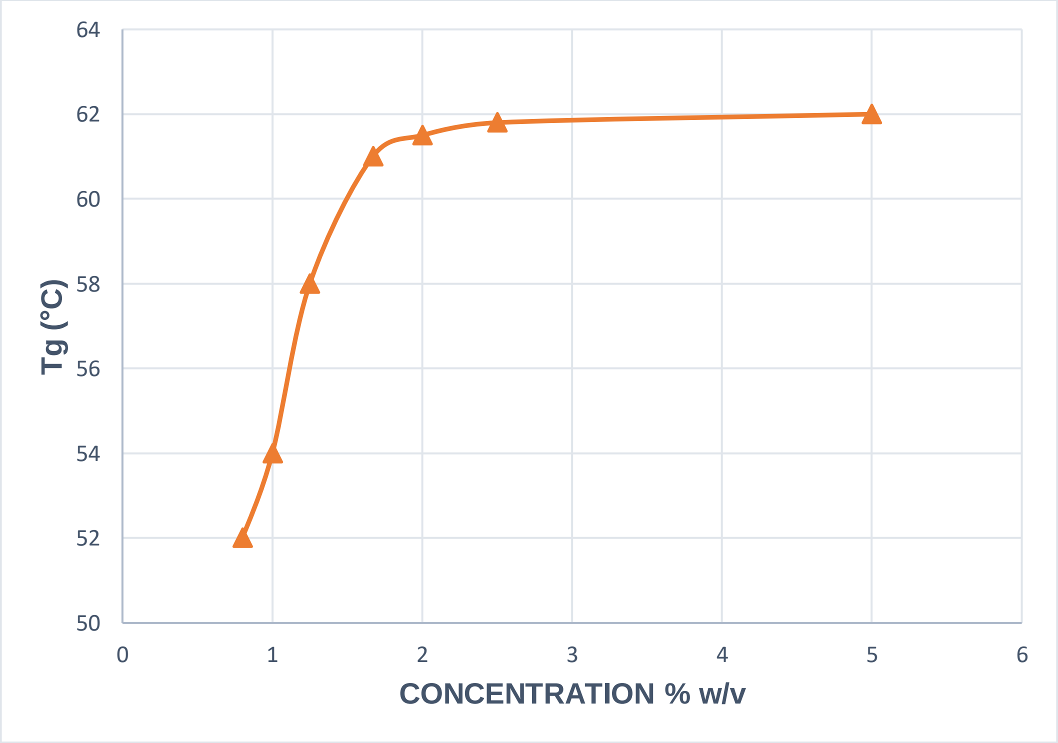

Figure S4. Reology. Dynamic strain sweep experiment of a hydrogel of **1** an DMSO gel of **2** at 25ºC.

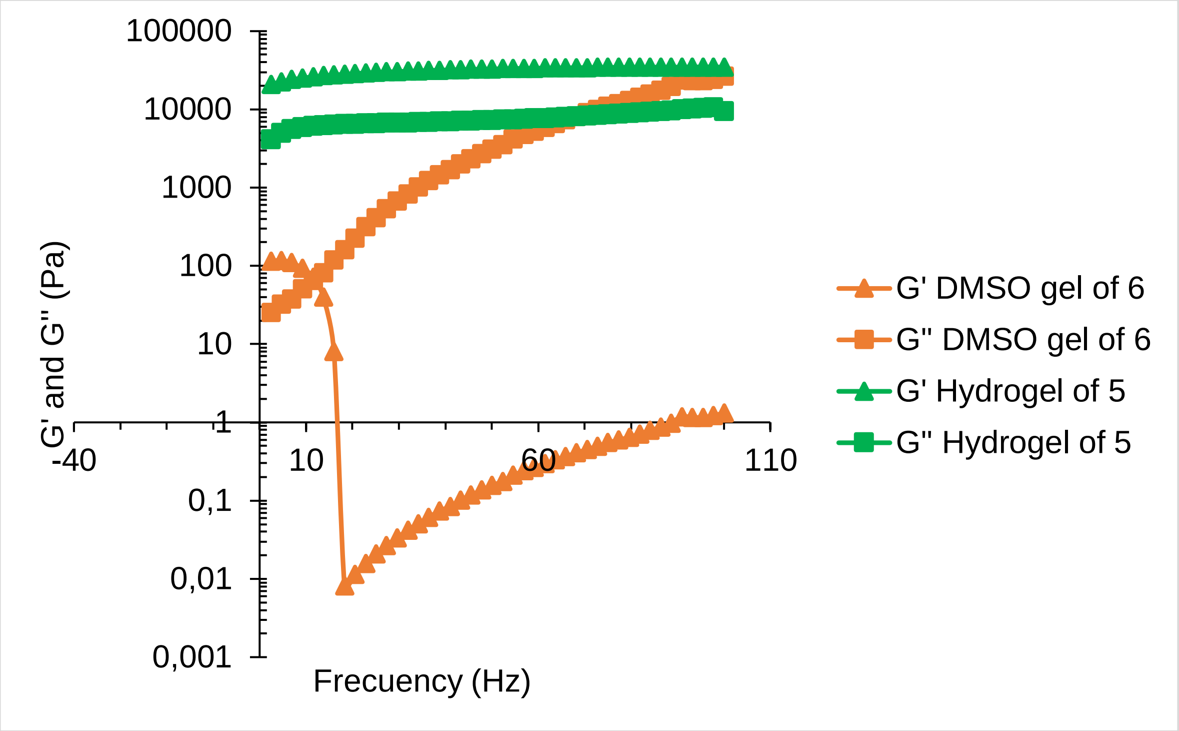

Figure S5. Powder XRD experiments

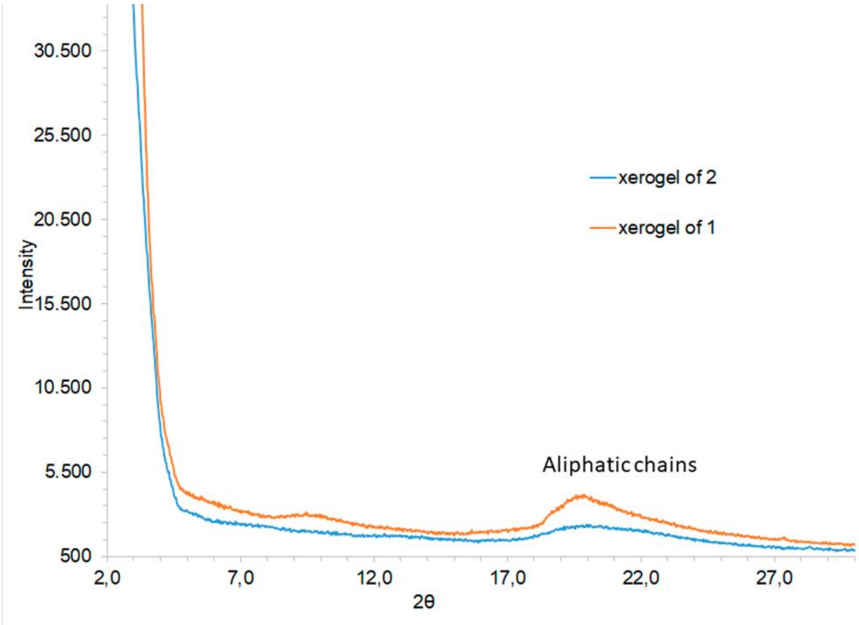

Figure S6. SEM images obtained from xerogels of a hydrogel of **1** (a) and a DMSO gel of **2** (b)

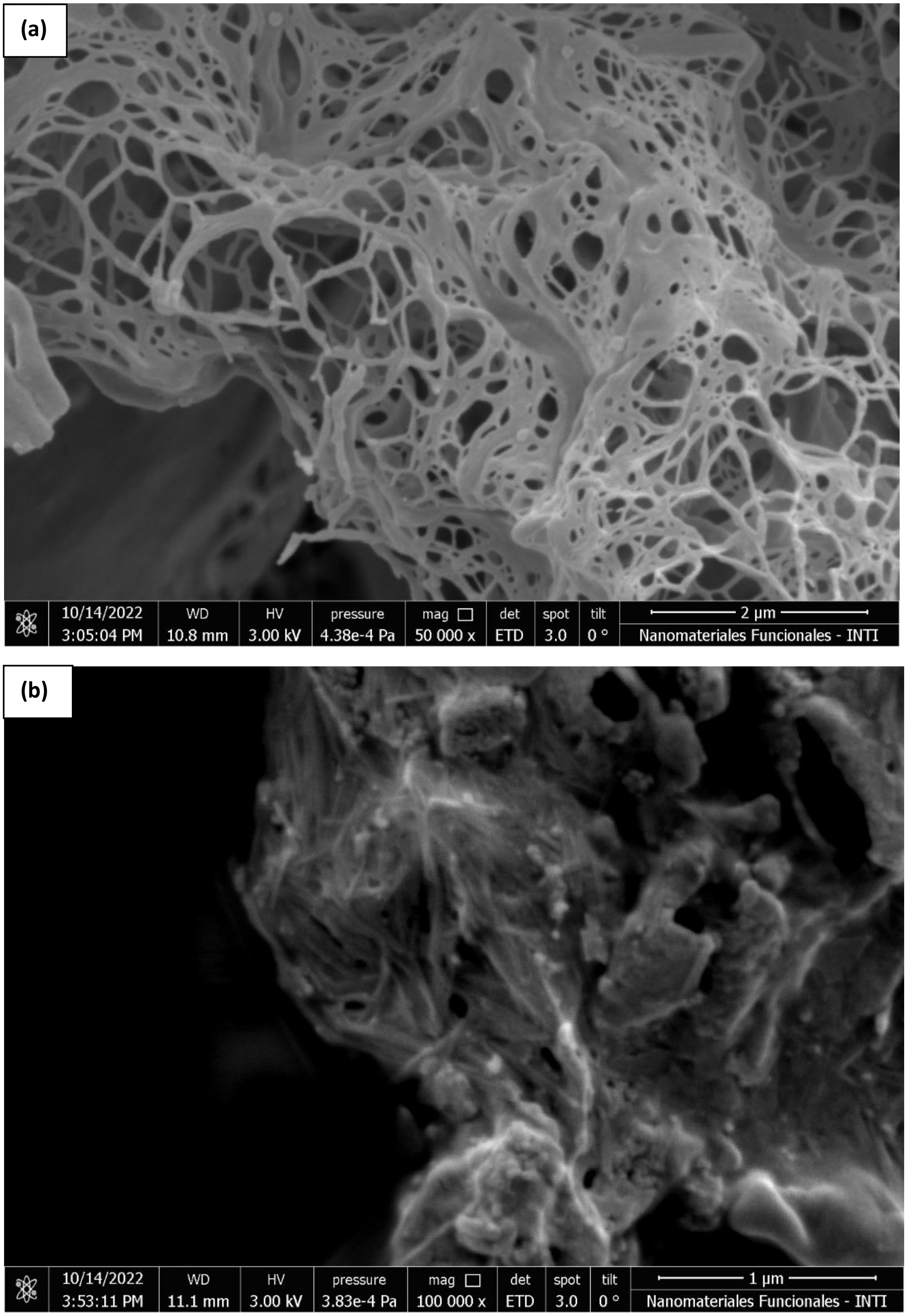

Figure S7. TEM image of the xerogel obtained from **2**

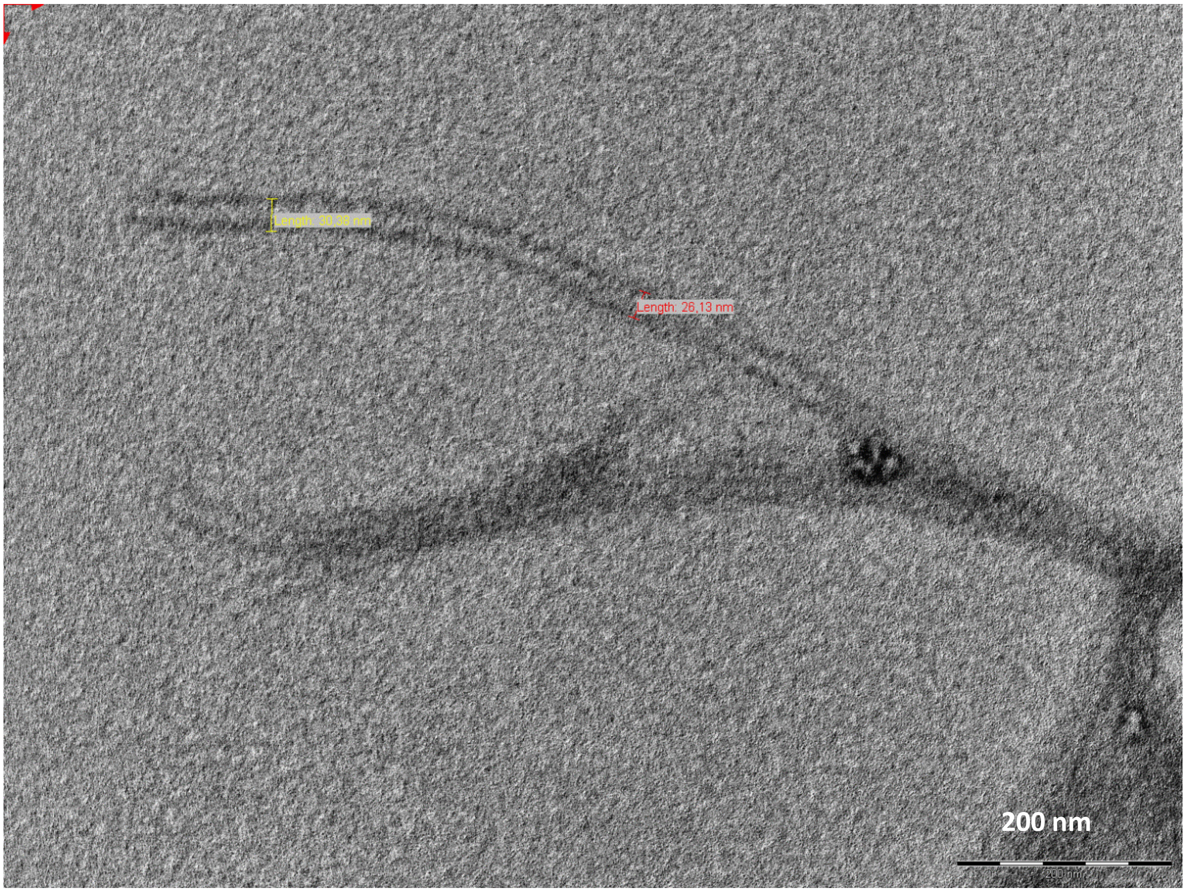

Supplement: Supplementary file 1 [file gels-10-00005-s001.zip › gels-2728906-supplementary.pdf]
